# Population dynamics of synthetic terraformation motifs

**DOI:** 10.1098/rsos.180121

**Published:** 2018-07-04

**Authors:** Ricard V. Solé, Raúl Montañez, Salva Duran-Nebreda, Daniel Rodriguez-Amor, Blai Vidiella, Josep Sardanyés

**Affiliations:** 1 ICREA-Complex Systems Lab, Universitat Pompeu Fabra, Dr Aiguader 88, 08003 Barcelona, Spain; 2 Institut de Biologia Evolitiva, CSIC-Universitat Pompeu Fabra, Passeig Marítim 37, 08003 Barcelona, Spain; 3 Santa Fe Institute 1399 Hyde Park Road, Santa Fe, NM 87501, USA; 4 Centre de Recerca Matemàtica, Edifici C, Campus de Bellaterra, 08193, Bellaterra, Barcelona, Spain; 5 Barcelona Graduate School of Mathematics (BGSMath), Edifici C, Campus de Bellaterra, 08193, Bellaterra, Barcelona, Spain; 6 Physics of Living Systems, Department of Physics, Massachusetts Institute of Technology, Cambridge, MA, USA

**Keywords:** catastrophic shifts, climate change, ecological engineering, synthetic ecology, mutualism

## Abstract

Ecosystems are complex systems, currently experiencing several threats associated with global warming, intensive exploitation and human-driven habitat degradation. Because of a general presence of multiple stable states, including states involving population extinction, and due to the intrinsic nonlinearities associated with feedback loops, collapse in ecosystems could occur in a catastrophic manner. It has been recently suggested that a potential path to prevent or modify the outcome of these transitions would involve designing synthetic organisms and synthetic ecological interactions that could push these endangered systems out of the critical boundaries. In this paper, we investigate the dynamics of the simplest mathematical models associated with four classes of ecological engineering designs, named *Terraformation motifs* (TMs). These TMs put in a nutshell different ecological strategies. In this context, some fundamental types of bifurcations pervade the systems’ dynamics. Mutualistic interactions can enhance persistence of the systems by means of saddle-node bifurcations. The models without cooperative interactions show that ecosystems achieve restoration through transcritical bifurcations. Thus, our analysis of the models allows us to define the stability conditions and parameter domains where these TMs must work.

## 1. Introduction: terraforming the biosphere

All around the planet, ecosystems appear to be experiencing serious threats associated with climate change along with other human-driven impacts [1–4]. The current environmental footprint of humankind is not sustainable considering the limited natural resources and assimilation capacity of the Earth [5]. Other planetary boundaries such as climate change, loss of biosphere integrity, land-system change and altered biogeochemical cycles are now being crossed as a result of human activity [6,7]. More importantly, the pace of these responses to external changes is likely to be far from linear, and it has been suggested that it can actually involve tipping points given by discontinuous transitions [8,9].

Mounting evidence indicates that even apparently mild, but cumulative changes such as increased grazing, rising temperatures or decreased precipitation can trigger sudden shifts and ecological collapses [9–11]. These rapid changes, usually labelled as *catastrophic shifts* [12], typically involve sudden transitions from a given stable ecosystem to a degraded or even fully extinct state. This is the case for semi-arid ecosystems. They constitute more than 40% of the Earth’s land surface and are home of almost 40% of human population [13]. Global desertification is a major challenge for our biosphere: current predictions indicate that drylands will expand in the next decades, while some areas can experience rapid collapses. Here minor modifications in external parameters (such as grazing rate) can trigger a rapid decline into a desert state with bare, empty soil unable to sustain vegetation cover [14,15]. There is now a substantial understanding of past events associated with this type of rapid declines. Available data and models indicate that the termination of the green Sahara state about 5500 years ago was followed by a transition to another stable, alternative state [16]. The tipping point found here would then separate two potential attractors [17,18].

Tipping points are an unavoidable outcome of the intrinsic dynamics of ecosystems and societies [9,10,19,20]. Owing to the nonlinear nature of interactions among species within ecosystems and to the response functions associated with them, the existence of multistability (i.e. the presence of multiple stable states) is the rule, not the exception. For the same reason, most dynamical systems such as ecosystems can move from one state to another one through a catastrophic event. Shifts between alternative states are now known to be present in a broad range of situations and have been experimentally demonstrated in micro-, meso- and field scenarios [17,21,22]. Tipping points could have deep consequences for the outcome of the anthropogenic changes of our biosphere.

A possibility that involves active changes in ecosystems through the use of synthetic biology has been recently proposed [23–25]. This approach can be used, among other things, as a way to curtail the accumulation of greenhouse gases, remove or degrade plastic debris and other types of waste, act on phosphorus and nitrogen fixation, or slow down ecosystem degradation in arid and semi-arid ecosystems [24,26,27]. The key point of this proposal is that engineering living systems allows reaching large scales due to their intrinsic self-reproduction. This makes a big difference in relation to standard engineering schemes, where artefacts need to be fully constructed from scratch. Instead, once a designed micro-organism is released, appropriate conditions should allow the living machines to self-renew and to scale by themselves.

Terraforming—modifying non-habitable planets into Earth-like systems—is here adopted to refer to the restoration of degraded ecosystems. This approach needs ecological designs that guarantee yield, as well as a limited evolutionary potential. A clever usage of cooperative loops or habitat constraints can provide these bounds on the impact of synthetic organisms. In this paper, we propose four possible engineering motifs that can cope with these two constraints. We do not consider explicit case studies (i.e. detailed genetic constructs or designed organisms) but the logic design schemes.

As proposed in [23], a novel form of addressing the previous issues would be to design synthetic organisms capable of interacting in predefined ways with target species or substrates in ways that can prevent undesirable responses [24,28,29]. The main reason for such an approach is that synthetic organisms can be seen as some class of living machines that have been designed as such to perform specific functionalities. The two main characteristics of these microscopic living systems are (i) they exhibit evolutionary dynamics and thus can change over time as a consequence of mutations, gene transfer or gene loss, among others; and (ii) living machines replicate and are thus capable of expanding their populations to scales many orders of magnitude larger than the originally designed populations. Reversion to the wild-type, via gene loss, may be an expected process in many cases, particularly when dealing with microbes [30]. This phenomenon has been recently shown for RNA viruses, even when the introduced genes involved a fitness gain [31].

Regarding the impact, evolving strains can develop advantageous traits but these traits could have a negative impact on the host ecosystem. Thereby, this approach needs to consider not just the success of its new ecological function, but also the impact and the evolutionary potential. In this context, a rational design of either ecological interactions and habitat constraints should guarantee its efficiency limiting the impact and the evolvability of synthetic organisms. It was argued that some special classes of engineered ecological motifs might well provide such strategy [23,26]. We labelled these basic designs *Terraformation motifs* (hereafter TM).

**Figure 1.**
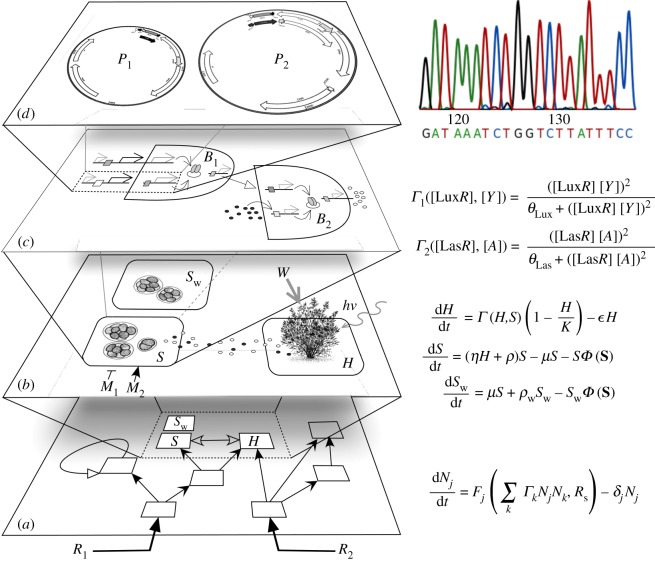
Multiple levels involved in the development of a theory of synthetic terraformation of ecosystems. Here the different scales are shown at the left and potential mathematical or formal descriptions at the right. Several layers of complexity need to be considered (in principle) including (*a*) whole population dynamics of ecosystems, with the population levels of each species described in terms of *N*_*j*_ variables. The time evolution of each of these variables would follow a deterministic or stochastic Lotka–Volterra formulation. A smaller scale level (*b*) considers the dynamics of a few given species, which can include synthetic candidates (here indicated as *S*) derived from a wild-type strain (here indicated as *S*_w_) and a plant host *H*. This small subset can be described, as a first approximation, by means of a small number of coupled equations, defining a three-species subgraph. At the species, cell level, the mathematical description of molecular interactions typically involves many coupled equations with nonlinear responses among genes and signalling molecules (here we just indicate a typical form of these cooperative interactions terms *Γ*_1_,*Γ*_2_). Finally, at the gene-sequence level, designed constructs (*d*) must be engineered in order to operate under predictable circumstances.

Our approach requires dealing with multiple scales, as summarized in figure 1. Here we display four levels of complexity: the ecological networks and the flow of resources within them (figure 1*a*); the specific nature of interactions among pairs of species including both wild-type and synthetic strains with their hosts (*H*), figure 1*b*; the required regulatory components, considering the cellular networks that will operate within cells (and how to engineer them, figure 1*c*); as well as the bottom level description where genetic sequences and available genetic toolkits are considered (figure 1*d*). In this paper, we approach these TMs from the point of view of their underlying population dynamics (figure 1*b*). We will consider the minimal mathematical models associated with each TM and determine the conditions for the survival or extinction of the synthetic organisms.

The aim of this paper is to make a few initial steps towards a general ecological theory for TMs seeking to understand the way these ecosystems might behave and how their populations will achieve different equilibria. By general theory, we mean the development of theoretical models able to explain the expected behaviours for TMs under different initial conditions and parameter values. Following the scheme outlined in figure 1, two important levels of complexity are presented when studying whole communities (figure 1*a*), and also when we consider a detailed description of species as cellular networks (figure 1*c*). As occurs with standard population dynamics, we need to start with the simple, few-species models indicated in figure 1*b*, somewhat averaging the details defining each particular partner at the smaller scale and also ignoring the multispecies nature of interaction defining the upper scale. As will be discussed below, such mesoscopic approach makes sense in the contexts discussed here.

All of the motifs, which are displayed in electronic supplementary material, Fig. S1, are explicit instances of the four basic classes of TMs presented in Solé *et al.* [26]. Electronic supplementary material, Fig. S1, also shows possible model organisms that could be used as candidates for their bioengineering. Specifically, in this article, we explore cooperative motifs with: *direct* (§2.2.1; electronic supplementary material, Fig. S1a) and *indirect* (§2.2.4; electronic supplementary material, Fig. S1b) cooperation. We also investigate two different restoration systems: *function-and-die* motif (§2.3.1; electronic supplementary material, Fig. S1c) and *sewage and landfill* motif (§2.3.2; electronic supplementary material, Fig. S1d). Before studying these particular motifs, we first introduce mean field models for the evolutionary and ecological process that are common to the four TMs (§2.1; electronic supplementary material, S1).

## 2. Results and discussion

### 2.1. Population dynamics of wild-type and synthetic strains under mutation

Let us first consider the simplest scenario tied to the TMs. It does not define a TM in itself, but it does contribute to an important piece of the underlying population dynamics. Here we consider the presence of two populations: the wild-type (hereafter WT) and the synthetic (hereafter SYN) strains. We assume that the SYN has been obtained by engineering the WT strain and that the SYN can revert to the WT by mutation or by the loss of the introduced genetic construct during the process of replication or during the life of the SYN.

#### 2.1.1. Wild-type strain–synthetic strain system with mutation

**Figure 2.**
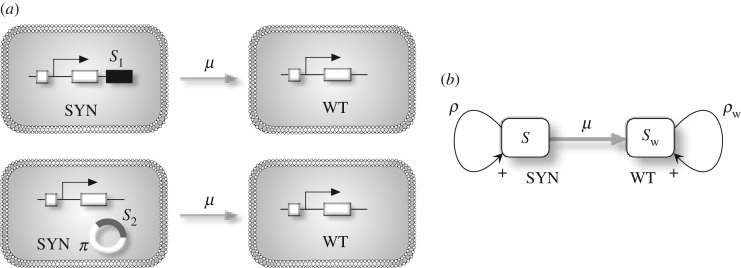
(*a*) Transitions from a synthetic (SYN) towards a wild-type (WT) strain can occur, for example, once the introduced constructs (either within the genome as *S*_1_ or as an external plasmid element *π*, indicated as *S*_2_) are lost. Such a loss of genetic material will typically occur at some rate *μ*. The basic scheme associated with this process is shown in (*b*). *S*_w_ and *S* are the population sizes of the WT and the SYN strains, with replication rates *ρ*_w_ and *ρ*, respectively.

First, we introduce a system with a population of SYN (*S*) and WT (*S*_w_) strains. Figure 2*a* displays schematically a manipulated organism (microbe) where a given gene has been modified, perhaps adding some additional synthetic constructs either inserted within the genome (such as *S*_1_) or within a plasmid *π* (indicated as *S*_2_). Either case, we simply assume that these constructs can be lost at a rate *μ*, giving place to the WT strain. The previous processes can be summarized using a transition diagram as shown in figure 2*b*, where the replication rates for the WT and the SYN strains are given by *ρ*_w_ and *ρ*, respectively. If nothing else is included, the dynamics for these two populations can be described by the following couple of linear differential equations:

$$\begin{array}{rl} \frac{dS}{dt} & =\rho (1-\mu )S,\\ \frac{dS_{w}}{dt} & =\rho_{w}S_{w}+\mu \rho S. \end{array}$$

The previous model is rather simple and the time solutions can be obtained explicitly (see electronic supplementary material, Section 1.1). A key process that needs to be considered is competition, which introduces a first nonlinearity constraining the persistence of the SYN strain. The dynamics with competition is explored in the following section.

#### 2.1.2. Model with mutation and competition

**Figure 3.**
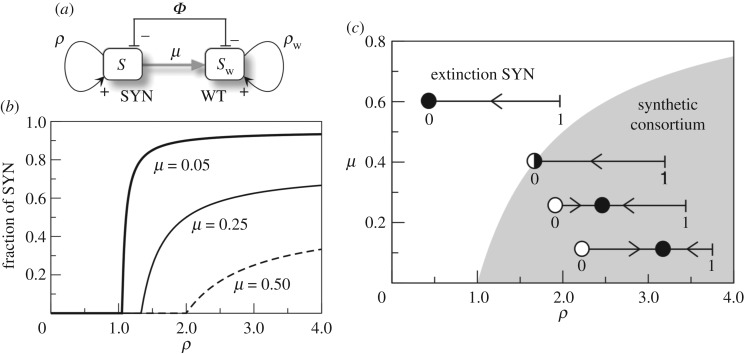
(*a*) Competition between wild-type (WT) and synthetic (SYN) strains, with population numbers *S*_w_ and *S*, respectively. (*b*) Transcritical bifurcation for three different values of *μ* at decreasing *ρ*, with *ρ*_w_=1 involving the extinction of the SYN strain at *ρ*=*ρ*_*c*_ (see electronic supplementary material, Section S1.2). (*c*) Phase diagram (*ρ*,*μ*) displaying persistence (grey) and extinction (white) of the SYN species. The dynamics in one-dimensional phase space *S* are illustrated as the parameters (from bottom-right to upper-left) approach and cross the transcritical bifurcation value (black and white circles denote stable and unstable fixed points, respectively).

Let us consider a situation where we simply consider the two species, the WT and the SYN, competing for available resources. In figure 3*a*, we summarize the structure of their interactions. They compete (as shown by the mutual negative feedback) and, following the model introduced in the previous section, also replicate at rates *ρ* and *ρ*_w_, respectively. The system with competition can be described by

$$\begin{array}{rl} \frac{dS}{dt} & =\rho (1-\mu )S-S\Phi (\text{S}),\\ \frac{dS_{w}}{dt} & =\rho_{w}S_{w}+\mu \rho S-S_{w}\Phi (\text{S}). \end{array}$$

Competition is introduced assuming a constant population (CP) constraint by means of the outflow term *Φ*(**S**)=*Φ*(*S*,*S*_w_) (this term will be used in all of the following models). The CP assumption captures the competition of the WT and the SYN in the same ecological niche. As mentioned, when a given microbe replicates, daughter cells might lose the gene constructs introduced in the engineering process. Such scenario should be expected (and occurs often in experimental conditions) if the fitness advantage of the synthetic organism does not compensate for the metabolic burden associated with the maintenance of additional genetic information.

Assuming a CP, the previous equations can be collapsed to one by considering *S*+*S*_w_=1 and $\dot{S}+{\dot{S}}_{w}=0$, having

2.1 $$\frac{dS}{dt}=(\rho -\rho_{w})S(1-S)-\mu \rho S,$$

which is formally equivalent to a SIS-like model of epidemic spreading [32]. The dynamics of this system is summarized in figure 3 (see electronic supplementary material, Section 1.2 for details). Equation (2.1) has two fixed points: S^*^_0_=0 (extinction of the SYN strain) and S^*^_1_=(1−(*μρ*)/(*ρ*−*ρ*_w_)). This system, which behaves like the logistic equation with density-independent degradation, suffers a transcritical bifurcation, as shown in figure 3*b* as a function of parameter *ρ*, or by means of a two-parameter bifurcation diagram (figure 3*c*; see also the associated phase space diagrams). The mutation bifurcation value is given by *μ*_*c*_=1−(*ρ*_w_/*ρ*). Note that replication rates for the WT and SYN can also act as bifurcating parameters.

In the model above, we considered that the processes of mutation or gene loss were tied to the process of replication. However, the genetic construct could be modified or lost during the lifetime of the SYN strain, not necessary during cells’ duplication. A model taking into account this process also undergoes a transcritical bifurcation similar to the model previously discussed (see electronic supplementary material, Section 1.2, for details on fixed points and bifurcation values for this model).

### 2.2. Mutualistic terraformation motifs

**Figure 4.**
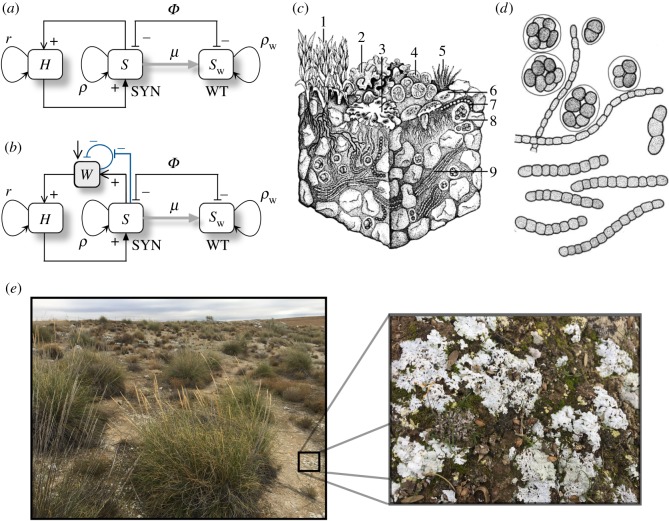
Terraformation motifs involving cooperation among synthetic (SYN) engineered micro-organisms and multicellular hosts (*H*). The SYN can revert to the WT either by mutation or by losing the engineered construct at a rate *μ*. Two motifs involving direct (*a*) and indirect (*b*) positive interactions among both partners defining a mutual dependency are explored. One potential scenario for this class is provided by dryland ecosystems, with plants being the hosts. In these habitats, the soil crust (*c*) provides a spatially, well-organized community of microbial species that can help engineering cooperative interactions. An engineered microbe capable of improving moisture retention can have a very strong effect on the underlying plant species. In soil crusts, a whole range of species adapted to water-poor conditions exist (drawing after Belnap & Lange [35]). Here we display: (1) mosses, (2,3) lichens, (4,5,7,9) cyanobacteria, (6) fungi and (8) green algae. A drawing of these species is shown in (*d*) exemplified by cells of *Nostoc* genus. (*e*) Soil crust surrounding isolated plants in a semi-arid ecosystem from central Spain. The enlarged view displays the detailed structure of the soil crust mainly composed of lichens and mosses.

A candidate organism to be engineered for modifying ecological systems should not be capable of decoupling itself from other species in such a way that becomes an expanding invader [33,34]. One especially appealing scenario is given by engineered mutualistic interactions (figure 4*a*,*b*).

In this context, cooperation has been successfully engineered experimentally in yeast [36] as well as in spatially extended populations of *Escherichia coli* [37]. Mutualism requires a double positive feedback where the synthetic species *S* benefits—and is benefited by—its host *H*. Ideally, design failure should end in the disappearance of the modified species. Because mutualism deals with two partners, the synthetic species will be constrained by the population of its mutualist partner and such a tight bond is especially convenient, as shown below.

Several targets can be conjectured. One particularly relevant class is given by the bacteria–root dependencies exhibited by plants [38] and particularly plant crops [39] with their surrounding microbiome. The main case study where this motif applies is provided by semi-arid ecosystems, already discussed in the Introduction section. In these ecosystems, a patchy vegetation cover is usually present, with species adapted to low moisture, extreme temperatures and high UV radiation.

A crucial component of these ecosystems is the biological soil crust (figure 4*c*–*e*), defining a complex living skin enclosed within a few centimetres of the topsoil [40,41]. Interactions between the soil crust and the vegetation have been modelled mathematically [42,43]. These are remarkable communities hosting a wide variety of species and largely mediating the energy and matter flows through the soil surface. They are known to help to preserve biodiversity and provide a reliable monitorization system for ecosystems’ health. In general, the more arid the environment the less diverse is the community, and, since plants and the biocrust are strongly related to each other, increased aridity leads to a smaller vegetation cover, less organic carbon reaching the soil, decreased micro-organism diversity, reduced plant productivity and loss of multifunctionality [44–46].

The basic schemes representing the interactions between the different components of the motif are shown in figure 4*a*,*b*. These are of course oversimplified pictures, since we ignore the multispecies composition of the biocrust. This simplification is done with the goal of understanding the behaviour displayed by minimal models, in the spirit of fundamental population dynamics models [47–49]. The first case (figure 4*a*) involves a direct impact through some tight relationship with the host plant, which can be, for example, an engineered symbiosis [50]. The second case (figure 4*b*) relies on an indirect cooperation mediated by the influence of the *S*_w_ species on, for example, moisture. Let us consider and analyse the two scenarios separately.

#### 2.2.1. Direct cooperation

The first type of cooperation motif deals with a synthetic strain that enhances the replication rate of the target (host) species (figure 2*a*). Here the best example would be to start from a free-living species and engineer it in order to build a new strain that becomes an obligate mutualist. Such transition has been shown to be possible and has been created by artificially forcing a strong metabolic dependence [51–54]. These systems have shown that the final product can be a physically tight interaction between the two partners [37].

This case study can be approached by a system of coupled differential equations as follows:

$$\begin{array}{rl} \frac{dH}{dt} & =\Gamma (H,S)\left(1-\frac{H}{K}\right)-\epsilon H,\\ \frac{dS}{dt} & =(\eta H+\rho )S-\mu S-S\Phi (\text{S}),\\ \frac{dS_{w}}{dt} & =\mu S+\rho_{w}S_{w}-S_{w}\Phi (\text{S}). \end{array}$$

In this model, we make the assumption that the two strains (SYN with state variable *S*; and WT with state variable *S*_w_) compete for available space and/or resources while the engineered strain is involved in a cooperative interaction with the host. Here *Γ*(*H*,*S*) is a growth function for the host (see below), *K* being its carrying capacity. Parameter *ϵ* is the density-independent death rate of the host. Constant *η* is the growth of strain *S* due to the mutualistic interaction with the host. The other parameters *ρ*, *ρ*_w_ and *μ* have the same meaning as in the previous sections. The function *Φ*(**S**) also stands for the outflow of the system, introducing competition. As we previously did, under the CP assumption, we can collapse the dynamical equations for the microbial strains into one, here with *Φ*(**S**)=*ηHS*+*ρS*+*ρ*_w_*S*_w_, and thus the equation for the synthetic population reads now

$$\frac{dS}{dt}=(\eta H+\rho -\rho_{w})S(1-S)-\mu S.$$

Here we consider *Γ*(*H*,*S*)=(*r*+*γS*)*H*, assuming that the host is capable of growing (at a rate *r*) in the absence of the microbial strains, whereas the term *γHS* stands for the cooperative interaction. Two possible cases are investigated: an extreme case with strict cooperation where plants can only grow with the presence of the SYN strain (with *r*=0, §2.2.2 below); and facultative cooperation where the host can grow autonomously as well as is benefitted from the interaction with the SYN strain (with *r*>0, §2.2.3 below).

#### 2.2.2. Strict cooperation

The first scenario considers strict cooperation, which requires that the host can only grow via cooperation (i.e. *r*=0). For this particular case, the system has four biologically meaningful equilibrium points, given by $P_{0}^{*}=(0,0)$, $P_{1}^{*}=(0,S_{1}^{*}=1-\mu /(\rho -\rho_{w}))$ and the pair $P_{\pm }^{*}=(H_{\pm }^{*},S_{\pm }^{*})$ (see electronic supplementary material, Section S2). The fixed point $P_{1}^{*}$ will be outside the positive (biologically meaningful) phase space when *μ*>*ρ*−*ρ*_w_. Under the condition *μ*=*ρ*−*ρ*_w_, the fixed points $P_{0}^{*}$ and $P_{1}^{*}$ will collide since $P_{1}^{*}{|}_{\mu =\rho -\rho_{w}}=P_{0}^{*}=(0,0)$. As we will show below, such condition involves a transcritical bifurcation between equilibria $P_{0}^{*}$ and $P_{1}^{*}$.

Let us now study the local stability of the fixed points $P_{0}^{*}$ and $P_{1}^{*}$ by means of linear stability analysis. Since the expression of the fixed points $P_{\pm }^{*}$ is cumbersome, the analytic derivation of the eigenvalues for these fixed points is rather difficult, and their stability character will be determined numerically by means of phase portraits representation (all of the numerical results presented in this article are obtained by means of the fourth-order Runge–Kutta method with a time step *δt*=0.1).

The stability of the fixed point $P_{0}^{*}$ is computed from the characteristic equation $|\text{J}(P_{0}^{*})-\lambda^{\left(0\right)}I|=0$, **J** being the Jacobian matrix of the system and *I* the identity matrix. The eigenvalues of this fixed point are given by $\lambda_{1}^{\left(0\right)}=-\epsilon$ and $\lambda_{2}^{\left(0\right)}=\rho -\rho_{w}-\mu$. The first eigenvalue is always negative, and thus the stability of this fixed point is given by *λ*_2_. Hence, the fixed point $P_{0}^{*}$ will be stable when *μ*>*ρ*−*ρ*_w_, a scenario under which the host and the SYN strain will become extinct.

Let us now characterize the stability of the second fixed point, $P_{1}^{*}$. This equilibrium point, if stable, involves the extinction of the host and the survival of the SYN strain. The eigenvalues computed from $|\text{J}(P_{1}^{*})-\lambda^{\left(1\right)}I|=0$ are given by

$$\begin{array}{rl} \lambda_{1}^{\left(1\right)} & =\gamma (\rho -\rho_{w}-\mu )-\epsilon ,\\ \lambda_{2}^{\left(1\right)} & =(2\mu -(\rho -\rho_{w}))(\rho -\rho_{w})-\mu . \end{array}$$

The equilibrium point $P_{1}^{*}$ will be stable if

$$\gamma <\frac{\epsilon }{\rho -\rho_{w}-\mu }\;\mathrm{and}\;\mu <\frac{{\left(\rho -\rho_{w}\right)}^{2}}{2(\rho -\rho_{w})-1}.$$

**Figure 5.**
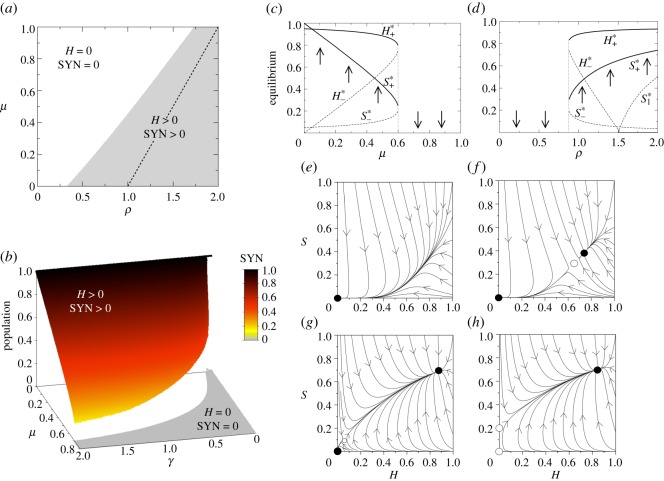
Dynamics of mutualistic TMs under strict cooperation with *r*=0, setting *ϵ*=0.05 and *η*=*K*=*ρ*_w_=1. (*a*) Regions in the parameter space (*ρ*,*μ*) where the host and the synthetic strains (SYN) survive (grey area) and become extinct (white area), using *γ*=0.5. The dashed line indicates the transcritical bifurcation and the boundary between the two states (grey and white) corresponds to the saddle-node bifurcation. (*b*) Equilibrium populations for the SYN strain in the space (*γ*,*μ*) with *ϵ*=0.05, *η*=*ρ*=*ρ*_w_=*K*=1. In (*c*) and (*d*) we display bifurcation diagrams tuning *μ* and *ρ*, respectively, using *ρ*=1 in (*c*) and *μ*=0.35 in (*d*). In both diagrams we set *γ*=0.5. Several phase portraits are displayed setting *μ*=0.4, *γ*=0.5 and (*e*) *ρ*=0.75; (*f*) *ρ*=0.91; (*g*) *ρ*=1.4; (*h*) *ρ*=1.6. Stable and unstable equilibria are indicated with black and white circles, respectively. The arrows indicate the direction of the orbits.

The previous results on the different fixed points and their stability nature are displayed in figure 5. First, we display the dynamics in the parameter spaces (*ρ*,*μ*) (figure 5*a*) and (*γ*,*μ*) (figure 5*b*). Here, for each pair of parameters we solved the system numerically plotting those parameter combinations where *H* and SYN persist at equilibrium (grey region). Here, there exists a frontier separating the grey and white zones that is given by a saddle-node bifurcation, which creates the pair of fixed points P^*^_+_ and P^*^_−_, which are a stable node and a saddle. These two equilibria are interior fixed points, and the stable node governs the survival of the host and the SYN strain.

The dashed line in the grey region of figure 5*a* separates two scenarios where the bifurcation between fixed points $P_{0}^{*}$ and $P_{1}^{*}$ takes place. In the grey region, the dynamics can be bistable, and the system can achieve persistence or extinction of the *H* and the SYN depending on the initial conditions and whenever the origin is stable. Figure 5*c*,*d* shows two bifurcation diagrams by tuning *μ* and *ρ*. The phase portraits of figure 5 display all possible dynamical scenarios with *H*–SYN extinction (figure 5*e*), *H*–SYN coexistence under bistability (figure 5*f*,*g*) and the *H*–SYN persistence without bistability, since after the bifurcation the origin becomes unstable and the node P^*^_+_ is a global attractor (figure 5*h*).

#### 2.2.3. Facultative reproduction and cooperation

The cooperative system considering facultative reproduction of the host (*r*>0) has five fixed points. Two of them are also given by P^*^_0_=(0,0), and $P_{1}^{*}=(0,S_{1}^{*}=1-\mu /(\rho -\rho_{w}))$ and $P_{\pm }^{*}=(H_{\pm }^{*},S_{\pm }^{*})$ (with *r*>0; see electronic supplementary material, Section S2), as we found in the previous section. For this system a new fixed point is found, named $P_{2}^{*}=(K(r-\epsilon )/r,0)$. This new fixed point, if stable, will involve the persistence of the *H* and the extinction of the SYN.

Linear stability analysis reveals that the eigenvalues for the fixed point P^*^_0_ are $\lambda_{1}^{\left(0\right)}=r-\epsilon$ and $\lambda_{2}^{\left(0\right)}=\rho -\rho_{w}-\mu$. Hence, this equilibrium point will be stable provided *r*<*ϵ* and *μ*>*ρ*−*ρ*_w_. The stability of the fixed point P^*^_1_ is given by the eigenvalues

$$\begin{array}{rl} \lambda_{1}^{\left(1\right)} & =(\rho -\rho_{w})+\gamma (\rho -\rho_{w}-\mu )-\epsilon ,\\ \lambda_{2}^{\left(1\right)} & =(2\mu -(\rho -\rho_{w}))(\rho -\rho_{w})-\mu . \end{array}$$

The first eigenvalue will be negative when

$$\gamma <\frac{\epsilon -(\rho -\rho_{w})}{\rho -\rho_{w}-\mu },$$

while the second eigenvalue remains the same as for the strict cooperation model analysed above.

Also, the stability of the fixed point P^*^_2_ is determined from the sign of the eigenvalues, given by $\lambda_{1}^{\left(2\right)}=\epsilon -r$ and $\lambda_{2}^{\left(2\right)}=\eta K(1-\epsilon /r)+\rho -\rho_{w}-\mu$. This fixed point will be stable provided *r*>*ϵ* and

$$\eta <\frac{\rho_{w}-\rho +\mu }{K(1-\epsilon /r)}.$$

Notice that the stability of this equilibrium also depends on parameters *ρ*, *ρ*_w_, *μ*, *K*, and *ϵ*.

The stability of the fixed points $P_{\pm }^{*}$ is also characterized solving the equations numerically, as we did in the previous section. For the numerical study we will use (if not otherwise specified) a value of *r*=0.5>*ϵ*=0.05. By doing so we ensure that the fixed point $P_{0}^{*}$ (which, if stable, involves the extinction of both *H* and SYN) is unstable.

**Figure 6.**
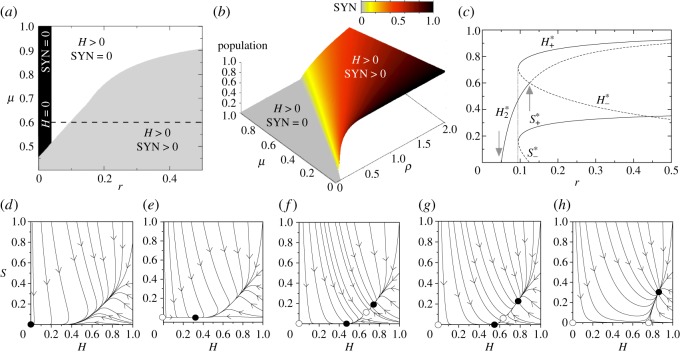
Dynamics and bifurcations for the mutualistic TMs with facultative reproduction of the *H* (*r*>0), using *ϵ*=0.05, *γ*=0.5, and *K*=*ρ*_w_=*η*=1. (*a*) Phase diagram (*r*,*μ*) computed numerically setting *ρ*=1. (*b*) Phase diagram (*ρ*,*μ*) also obtained numerically using *r*=0.5. (*c*) Bifurcation diagram using *r* as control parameter and fixing *μ*=0.6 and *ρ*=1 (the bifurcation diagram has been obtained tuning the values of *r* following the dashed line in panel (*a*)). Notice that at increasing *r* the system first suffers a transcritical bifurcation (first grey arrow), then a saddle-node bifurcation (vertical dotted line) and a second transcritical bifurcation (second grey arrow). Phase portraits representation with *μ*=0.6 and (*d*) *r*=0.045; (*e*) *r*=0.075; (*f*) *r*=0.0975; (*g*) *r*=0.1; and (*h*) *r*=0.2.

Figure 6 summarizes all the dynamical outcomes of this system. First, we display the equilibrium states of the system in the parameter spaces (*r*,*μ*) (i) and (*ρ*,*μ*) (ii) computed numerically. The space (*r*,*μ*) contains three different phases. For those values of *ϵ*>*r*, the outcome of the system is the extinction of both the *H* and the SYN (the black region in figure 6*a*), since the fixed point $P_{0}^{*}$ is stable. The transition to the scenario with *H* >0 and SYN =0 is governed by a transcritical bifurcation between the fixed points $P_{0}^{*}$ and $P_{2}^{*}$ (see phase portraits in figure 6*d*,*e*). After this bifurcation, the fixed point $P_{0}^{*}$ becomes unstable (white circle in the phase portraits of figure 6*e*–*h*) and $P_{2}^{*}$ stable (black circle).

Similar to the case for strict cooperation studied in the previous section, the boundary of the region where both *H* and SYN persist defines a saddle-node bifurcation responsible for the creation of the fixed points P^*^_+_ (node) and P^*^_−_ (saddle), involving bistability. Then, further increase of *r* makes the saddle point to collide with the fixed point $P_{2}^{*}$ in another transcritical bifurcation. Such a collision involves the interchange of stability between points P^*^_−_ and $P_{2}^{*}$. After the collision, the fixed point $P_{2}^{*}$ becomes unstable, and the saddle leaves the positive (biologically meaningful) phase space. Here, the stable node becomes asymptotically globally stable, bistability being replaced by monostability. Finally, figure 6*c* displays a bifurcation diagram using *r* as a control parameter. Note that this bifurcation diagram corresponds to the values of parameter *r* displayed with the horizontal dashed line in figure 6*a* (setting *μ*=0.6). Here one can follow the series of bifurcations discussed above.

#### 2.2.4. Indirect cooperation

As previously discussed, one of the most obvious candidates to apply the approach taken here is provided by semi-arid ecosystems and other water-controlled habitats where soil water interacts with a diverse range of soil and community properties, including carbon assimilation, transpiration rates or biomass production [55]. The biological soil crust is composed by a network of mutualists [56] and provides the ecological context suitable for vascular plants (figure 5*e*). It strongly influences key ecosystem processes and its diverse composition offers multiple opportunities for engineering cooperative loops.

Specifically, we aim at describing the impact of an engineered strain capable of improving water retention in the biocrust. This is illustrated by the production of extracellular polysaccharides by cyanobacteria [57–59], which have been shown to affect hydrological soil properties, as well as other important features such as soil carbon and maintenance of structural soil integrity [60]. These and other molecules (from vitamins to phytohormones) have been recognized to play a key role in helping plant growth and development [61].

The potentially beneficial role of increased extracellular molecules has been exploited in field experiments in desert habitats and sustainable agriculture under adverse ecological and edaphic conditions [62] through the direct addition of polysaccharides [63], cultivating outdoors combinations of cyanobacteria and plants [64], and massive inoculation of selected microbial strains [65]. Several positive results have been reported from these studies, suggesting a major role played by ecological interactions connecting molecular, cellular and population responses.

A minimal model that encapsulates the indirect cooperative interactions (see electronic supplementary material, Fig. S1b) involving water (state variable *W*) is provided by the following set of equations:

$$\begin{array}{rl} \frac{dH}{dt} & =\Xi (W,H)\left(1-\frac{H}{K}\right)-\epsilon H,\\ \frac{dS}{dt} & =(\eta H+\rho )S-\mu S-S\Phi (\text{S}),\\ \frac{dS_{w}}{dt} & =\mu S+\rho_{w}S_{w}-S_{w}\Phi (\text{S}),\\ \frac{dW}{dt} & =a-F(W,S)-\psi WH. \end{array}$$

The function *Ξ*(*W*,*H*)=*βψWH* is the growth rate of the host, which depends on the availability of water. Here, the constant *β* is the growth rate of the *H* depending on the water, while *ψ* is the fraction of water used by the *H* to grow. The population of the host has a logistic growth restriction with carrying capacity *K*, and a density-independent death rate, *ϵ*. Concerning the dynamical equation for the SYN strain, constant *η* is the growth rate of the *S* tied to the cooperation with *H*, *ρ* is the replication rate of the *S*, *ρ*_w_ is the replication rate of the WT and *μ* is the rate of gene loss or mutation. Now, the dilution flow is given by *Φ*(**S**)=(*ηH*+*ρ*)*S*+*ρ*_w_*S*_w_.

Following Klausmeier [66], the last equation includes three terms in the RHS: (i) a constant water input, *a* (rate of precipitation); (ii) water loss; and (iii) a term of water consumption by the vegetation. The function of water loss reads

$$F(W,S)=\frac{LW}{1+(S/S_{c})},$$

*L* being the maximum evaporation rate and *S*_*c*_ the rate of inhibition of the evaporation due to the presence of *S*. This function introduces a specific modulation by means of an inhibition function which includes both the term *LW* as well as a nonlinear decay associated with the presence of the synthetic population, which is capable of reducing water loss.

As we did for the previous models, we can reduce the system by using the linear relation *S*_w_=1−*S*, now having

$$\begin{array}{rl} \frac{dH}{dt} & =\beta \psi WH\left(1-\frac{H}{K}\right)-\epsilon H,\\ \frac{dS}{dt} & =(\eta H+\rho -\rho_{w})S(1-S)-\mu S,\\ \frac{dW}{dt} & =a-\frac{LW}{1+S/S_{c}}-\psi WH. \end{array}$$

For the sake of simplicity, we will use $\tilde{\rho }=\rho -\rho_{w}$. This system has five different fixed points, $P_{1\ldots 5}^{*}$, with

$$\begin{array}{rl} P_{1}^{*} & =\left(H=0,S=0,W=\frac{a}{L}\right),\\ P_{2}^{*} & =\left(H=0,S=1-\frac{\mu }{\tilde{\rho }},W=\frac{a(S_{c}\tilde{\rho }+\tilde{\rho }-\mu )}{S_{c}\tilde{\rho }L}\right),\\ P_{3}^{*} & =\left(H=\frac{K(a\beta \psi -L\epsilon )}{(a\beta +K\epsilon )\psi },S=0,W=\frac{a\beta +K\epsilon }{\beta L+\beta K\psi }\right) \end{array}$$

and two more fixed points $P_{4}^{*}$ and $P_{5}^{*}$ (see electronic supplementary material, Section S3, for the values of these fixed points and the Jacobian matrix). The eigenvalues for the fixed point $P_{1}^{*}$ are given by

$$\lambda_{1}=\frac{a\beta \psi }{L}-\epsilon ,\;\lambda_{2}=\tilde{\rho }-\mu \;\mathrm{and}\;\lambda_{3}=-L,$$

while the eigenvalues for $P_{2}^{*}$ are

$$\lambda_{1}=\frac{a\beta \psi }{LS_{c}}\left(1+S_{c}-\frac{\mu }{\tilde{\rho }}\right),\;\lambda_{2}=\mu -\tilde{\rho }\;\text{and}\;\lambda_{3}=LS_{c}\tilde{\rho }/(\mu -\tilde{\rho }(1-S_{c})).$$

**Figure 7.**
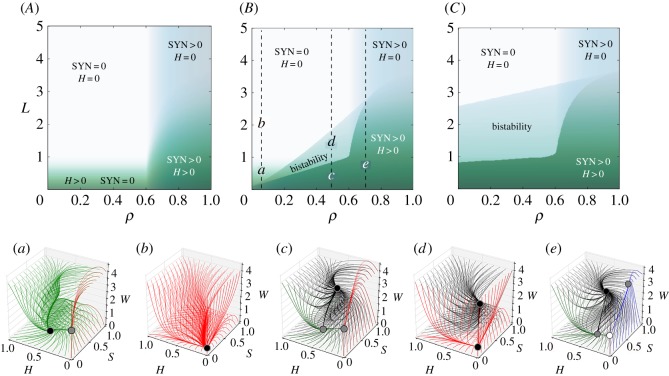
Dynamics for the indirect cooperation motif setting: *ψ*=*ϵ*=0.5, *β*=*K*=*a*=1 and *μ*=0.1. (Upper) Phase diagrams displaying the equilibrium for the vegetation (*H*, green gradient) and the synthetic strain (SYN, blue gradient) in the parameter space (*ρ*, *L*) with *S*_*c*_=0.3 and *ρ*_w_=0.5, using three values of *η*: (*A*) *η*=0; (*B*) *η*=1; and (*C*) *η*=5. The intensity of the colours for *H* and SYN is the sum of the equilibria starting from two different initial conditions (one near to 1 and one near to 0). The vertical dashed lines in panels *B* and *C* indicate the parameter ranges used in the bifurcation diagrams displayed in electronic supplementary material, Fig. S3. (Lower) Three-dimensional phase portraits corresponding to the parameter values indicated with small letters in panel (*B*) corresponding to the vegetated state (*a*); desertification (*b*); coexistence in a vegetated state (*c*); and engineered bistable ecosystem (*d*). In case (*e*) the SYN strain can survive without the vegetation, and the vegetation can survive without the SYN strain. The stability of the fixed points is indicated with different colours: stable (black), stable in a plane (grey) and only stable in one direction (white). The colours of the orbits indicate which states are achieved by the trajectories: desert (red), vegetated state (green), synthetic and desert (blue), and coexistence between *H* and SYN (black).

Relevant parameters that could be engineered are the replication efficiency of the SYN (*ρ*), the benefit the SYN obtains from the host (*η*), and the evaporation inhibition due to the action of the SYN microbia (*S*_*c*_). Figure 7 displays two-parameter phase diagrams, where the different dynamical scenarios can be visualized. Synthetic organisms shall have a higher expression load due to the SYN construct. This load would make the SYN grow slower than the WT (*ρ*<*ρ*_w_). In order to counterbalance this effect and make the SYN strain able to survive, the SYN can take advantage of the vegetation. If there is no symbiosis (*η*=0) the SYN organism only survives provided $\tilde{\rho }>\mu$ (figure 7*A*). For *η*>0, a bistable region exists and it becomes larger as the strength of symbiosis increases. The SYN survives even for *ρ*=0 if the symbiosis strength is 1 (figure 7*B*). For higher *η* values the vegetation survives for large evaporation rates (*L*), even with a low replication rate (figure 7*C*).

**Figure 8.**
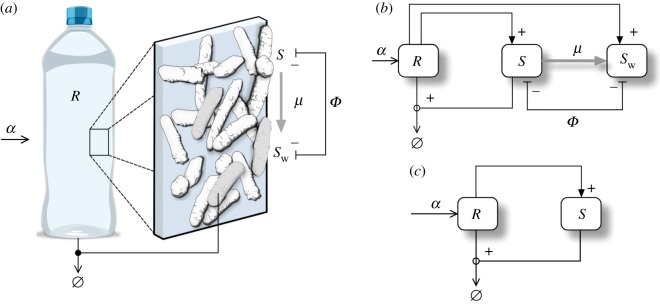
Terraformation motifs for the *function-and-die design*. This scheme applies to a diverse range of possible targets, such as marine plastic debris (*a*) where plastic is the resource, entering the system at a rate *α* and spontaneously degraded but also actively degraded by the microbial strains *S* and *S*_w_ which appear to be supported by the substrate. The formal motif diagram is shown in (*b*). In (*c*), we display a simplified motif where the synthetic strain has not been derived from a wild-type variant.

For a semi-arid ecosystem with a given fraction of vegetated area, the increase in the evaporation rate (e.g. increase in temperature) involves the full desertification of the system (see the changes between the phase portraits displayed in figure 7*a*,*b*). This process and the associated changes in the topology of the phase space can be visualized in electronic supplementary material, video S1. If evaporation increases in the engineered ecosystem without changing the replication rate, there is a region of bistability, and a saddle-node bifurcation is achieved (see electronic supplementary material, video S2). If $\tilde{\rho }$ is high enough the fixed point $P_{4}^{*}$ collides with $P_{2}^{*}$ in a bifurcation (this collision can be seen in electronic supplementary material, video S3). Once the system is optimally engineered, the vegetation can survive even if *ρ*<*ρ*_w_ and the evaporation is much higher (figure 7*d*). If $\tilde{\rho }<\mu$, the SYN will not be able to survive alone (figure 7*c*; electronic supplementary material, Fig. S2a), otherwise the engineered organism will survive even without vegetation (figure 8*e*; electronic supplementary material, Fig. S2f).

The engineering of the system can change the dynamics and the vegetation–desert transition from a transcritical bifurcation (involving $P_{3}^{*}$ and $P_{1}^{*}$) to a saddle-node bifurcation (where both $P_{4}^{*}$ and $P_{1}^{*}$ collide). This process can be seen in the bifurcation diagrams of electronic supplementary material, Fig. S3. The saddle-node bifurcation involves the emergence of bistability. If *ρ* is high enough the saddle-node bifurcation takes place after the transcritical bifurcation between the points $P_{3}^{*}$ and $P_{1}^{*}$. In this scenario, the re-vegetation will take place if the evaporation decreases (electronic supplementary material, Fig. S3e). The two stable states are given by: the coexistence of the *H*, *S* and *W*; and, depending on $\tilde{\rho }$, the desert alone (figure 7*d*) or the coexistence of both SYN and *W* (electronic supplementary material, Fig. S2g).

Another relevant parameter to be modified by the engineering of the SYN strain is *S*_*c*_. This parameter is related to the effect in the evaporation of water depending on the amount of SYN. When the threshold *S*_*c*_ is low, the amount of SYN population needed to have a positive effect in the environment is very low. This means that with a small fraction of the SYN the system could support a vegetated state. The SYN strain could be able to produce, for example, a large amount of polysaccharides capable of retaining water. The rate of production will be closely related to the inverse of the threshold. Depending on the difference between the replication efficiency ($\tilde{\rho }$) the region of bistability changes. If $\tilde{\rho }<\mu$ the bistability region is broader (electronic supplementary material, Fig. S2A), and if $\tilde{\rho }>\mu$ (electronic supplementary material, Fig. S2B) the coexistence state is broader. However, the limit where the saddle-node bifurcation takes place does not change (electronic supplementary material, Fig. S2).

### 2.3 Restoration motifs

One of the outputs of the Anthropocene is the accumulation of waste. In order to face this problem, we propose and investigate two restoration motifs given by the: *function-and-die* and *sewage and landfill* motifs.

#### 2.3.1 Function-and-die motifs

The TMs do not necessarily need to act within natural ecosystems. An alternative scenario, to be considered here and in the next section, is to take advantage of extensive wasteland habitats that have been created by humans and in some sense are already ‘synthetic’. These synthetic habitats offer opportunities for using bioremediation (including removal of undesired molecules), but can also act as novel substrates that can host useful synthetic micro-organisms.

A synthetic strain could use this substrate as a physical surface allowing it to grow and perhaps disperse. Oceanic plastic debris is an example of this situation (figure 8*a*). Plastics have been entering oceans and concentrating into large garbage gyres [67] since the 1950s at a rapid and global scale, leading to the generation of the so-called plastisphere [68,69]. This widespread class of anthropogenic waste is made of non-natural macromolecular structures that were not present in nature and thus there were no biological mechanisms expected at that time to degrade it. However, mounting evidence indicates that some species of micro-organisms have been adapted to this special class of substrate, effectively degrading it (see [70] and references therein) or at least contributing to its fragmentation and decay.

The TM analysed here takes its name from an intrinsically relevant property that defines an ecological firewall. The key idea is illustrated by a specific example that has been developed with the goal of repairing (self-healing) concrete cracks, which are a major challenge for the maintenance of infrastructures. The alkaline environment makes it difficult for most species to thrive but some species can be used for this purpose. Since repair requires filling a given volume (something that living things can do by reproducing themselves) and do it by means of a suitable material (and bacteria can do that too) synthetic biology appears as a potentially useful approximation here [71].

A microbe can be designed to grow and replenish cracks with calcium carbonate along with a secreted macromolecule that merges into a strong material [72,73]. A major advantage of this problem is that anaerobic bacteria are not going to survive outside the crack and thus selection immediately acts once the task is finished: the function (repair) is done and afterwards the synthetic strain is unable to survive. The right combination of genetic design and ecological constraints create a powerful safeguard.

More generally, we consider here the potential conditions for survival of a synthetic strain living on a given substrate that enters the system and is degraded. The synthetic strain can just degrade the resource or can additionally perform some given functionality. Since removal of plastic debris might actually be part of the goal, it might be unnecessary to use existing species associated with this substrate. Instead, it could be more efficient to simply design or evolve a highly efficient species capable of attaching to the plastic surface, being also able to outcompete other present species.

The mathematical model associated with the function-and-die motif (see electronic supplementary material, Fig. S1c) is given by

$$\begin{array}{rl} \frac{dR}{dt} & =\alpha -\delta R-\sigma RS,\\ \frac{dS}{dt} & =\eta \sigma SR-\mu S-S\Phi (\text{S}),\\ \frac{dS_{w}}{dt} & =\rho_{w}S_{w}+\mu S-S_{w}\Phi (\text{S}). \end{array}$$

The state variables for this model are the resource (*R*) and both the SYN (*S*) and the WT (*S*_w_) strains. Here *α* is the constant income rate of the resource (e.g. plastic), *δ* is the spontaneous degradation rate of the reource and *σ* is the elimination rate of the resource due to the action of the SYN species. Additionally, *η* is the growth rate of the SYN strain associated with the degradation of the resource (*μ* and *ρ*_w_ have already been defined).

For this model, the outflow term is given by *Φ*(**S**)=*ησSR*+*ρ*_w_*S*_w_. Assuming again a CP constraint, *S*+*S*_w_=1, the system can be reduced to the following model:

$$\begin{array}{rl} \frac{dR}{dt} & =\alpha -\delta R-\sigma RS,\\ \frac{dS}{dt} & =S[(1-S)(\eta \sigma R-\rho_{w})-\mu ]. \end{array}$$

This system has three fixed points, given by

$$P_{0}^{*}=\left(R_{0}^{*}=\frac{\alpha }{\delta },S_{0}^{*}=0\right)$$

(i.e. the only-plastic system) and the pair P^*^_1,2_=(R^*^_1,2_,S^*^_1,2_). The coordinates of the fixed point P^*^_1_=(R^*^_1_,S^*^_1_) are given by

$$R_{1}^{*}=\frac{\alpha \eta \sigma +\rho_{w}(\delta +\sigma )+\sigma \mu -\sqrt{\Psi }}{2(\delta +\sigma )\eta }$$

and

$$S_{1}^{*}=\frac{\alpha \eta \sigma -\rho_{w}(\delta -\sigma )+\sigma \mu +\sqrt{\Psi }}{2\sigma \eta_{w}}$$

with *Ψ*=−4*α*(*δ*+*σ*)*ηη*_w_+(*αη*+*δη*_w_+*σ*(*ρ*_w_+*μ*))^2^. The coordinates of the fixed point P^*^_2_=(R^*^_2_,S^*^_2_) read like the coordinates above but with a change of sign, with

2.2 $$R_{2}^{*}=\frac{\alpha \eta \sigma +\rho_{w}(\delta +\sigma )+\sigma \mu +\sqrt{\Psi }}{2(\delta +\sigma )\eta }$$

and

2.3 $$S_{2}^{*}=\frac{\alpha \eta \sigma -\rho_{w}(\delta -\sigma )+\sigma \mu -\sqrt{\Psi }}{2\sigma \eta_{w}}.$$

Numerical results obtained for this model suggested that the coordinate $S_{1}^{*}>1$ within the range 0≤*η*≤1. For this case, the fixed point $P_{1}^{*}$ is outside the simplex and it is not biologically meaningful (recall that the CP constraint assumes that *S*_w_+*S*=1, and thus $S_{1}^{*}$ cannot be higher than 1). Under this scenario, the dynamics in the interior of the simplex is governed by the fixed point $P_{2}^{*}$. In order to check whether the fixed point $P_{1}^{*}$ lives inside the simplex, we performed a simple numerical test. We computed the value of the coordinate $S_{1}^{*}$ for 10^10^ combinations of random parameters (with uniform distribution) within the ranges: *α*∈[0,50], and *δ*,*σ*,*η*,*ρ*_w_,*μ*∈[0,1]. For all these combinations, we obtained values of S^*^_1_>1.

The Jacobian matrix for this model reads

$$J(P_{k}^{*})={\left(\begin{array}{cc} -\delta -S\sigma & -\sigma R\\ S\eta \sigma (1-S) & (R\eta \sigma -\rho_{w})(1-2S)-\mu \end{array}\right)}_{P_{k}^{*}}.$$

From $\det|J(P_{0}^{*})-\lambda I|=0$, we compute the associated eigenvalues for *P*_0_, given by

$$\begin{array}{rl} \lambda_{1} & =-\delta ,\\ \lambda_{2} & =\frac{\alpha }{\delta }\eta \sigma -\rho_{w}-\mu . \end{array}$$

We note that *λ*_1_ is always negative and thus the stability of P^*^_0_ will entirely depend on *λ*_2_. The change of stability of this point can be computed from *λ*_2_=0. The critical values of the parameters in *λ*_2_ that involve a change of sign of this eigenvalue are

$$\begin{array}{rl} \mu_{c} & =\frac{\alpha }{\delta }\eta \sigma -\rho_{w},\;\eta_{c}=\frac{(\rho_{w}+\mu )\delta }{\alpha \sigma },\;\rho_{w}^{c}=\frac{\alpha }{\delta }\eta \sigma -\mu ,\\ \alpha_{c} & =\frac{(\rho_{w}+\mu )\delta }{\eta \sigma }\;\mathrm{and}\;\delta_{c}=\frac{\alpha \eta \sigma }{\rho_{w}+\mu }. \end{array}$$

Following the previous critical conditions, the fixed point P^*^_0_ will be unstable (i.e. saddle-point with *λ*_2_>0, meaning that the SYN strain will survive) when *μ*<*μ*_*c*_, *η*>*η*_*c*_, $\eta_{w}<\eta_{w}^{c}$, *α*>*α*_*c*_ or *δ*<*δ*_*c*_. For example, at *μ*=*μ*_*c*_, both fixed points $P_{0}^{*}$ and $P_{2}^{*}$ collide since

$$P_{0}^{*}{|}_{\mu =\mu_{c}}=P_{2}^{*}{|}_{\mu =\mu_{c}}=\left(\frac{\alpha }{\delta },0\right).$$

At the bifurcation value, these fixed points also interchange stability. Hence, a transcritical bifurcation is found for this motif. The same behaviour is found at *η*=*η*_*c*_, $\rho_{w}=\rho_{w}^{c}$, *α*=*α*_*c*_ and *δ*=*δ*_*c*_.

**Figure 9.**
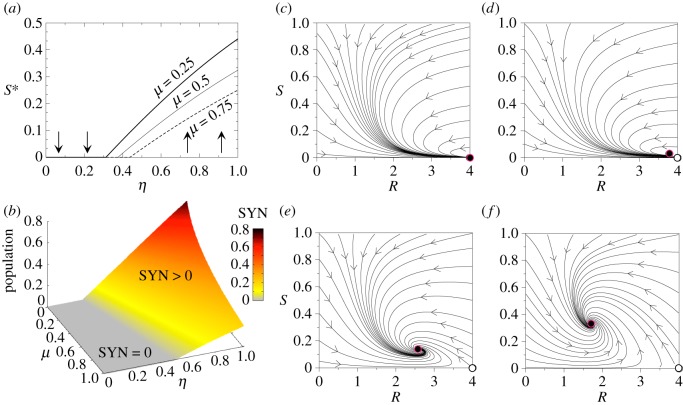
Bifurcations and dynamics of the function-and-die TM with competing microbes. In (*a*) we display the stationary population of the synthetic strain (S*) against the parameter *η* that weights the efficiency of the resource–microbe interaction. Here we fix *α*=*σ*=*ρ*_w_=1 and *δ*=0.25, using different values of the reversion parameter *μ*. We specifically use *μ*=0.25 (thick line), *μ*=0.5 (thin line) and *μ*=0.75 (dashed line). The vertical arrows indicate the stability of the fixed point P^*^_0_. (*b*) Survival and extinction phases in the parameter space (*μ*,*η*) computed numerically, using the same parameter values of (*a*). Several phase portraits are displayed setting *μ*=0.25 and (*c*) *η*=0.3<*η*_*c*_=0.3125 , (*d*) *η*=0.34>*η*_*c*_, (*e*) *η*=0.5 and (*f*) *η*=0.8. The internal stable fixed point (black circle highlighted in red) is the fixed point P^*^_2_. The fixed point $P_{1}^{*}$ is not biologically meaningful in the range analysed, since $S_{1}^{*}>1$ (see electronic supplementary material, Fig. S4). Unstable equilibria are indicated with white circles.

Some examples of the bifurcation diagrams associated with this model are shown in figure 9*a*,*b*. Here we represent the equilibrium populations S* (computed numerically) of the SYN strain against the efficiency parameter *η*. A continuous transition given by the transcritical bifurcation takes place for *η*=*η*_*c*_, when the SYN strain overcomes the competitive advantage of *S*_w_. Given the definition of *η*_*c*_, for a fixed input and degradation of the resource and mutation rate, the condition *η*>*η*_*c*_ is achieved once the advantages of the engineered strain overcome the growth rate of the WT.

An interesting feature of this diagram is that, even for large values of the reversal parameter *μ* we obtain high population values provided that *η* is large enough. The changes in the phase space at increasing *η* are displayed in figure 9*c*–*f*. In figure 9*c* the fixed point $P_{0}^{*}$ is globally asymptotically stable (here *η*<*η*_*c*_). Once *η*>*η*_*c*_ (figure 9*d*–*f*), the fixed point $P_{2}^{*}$ enters into the phase plane having exchanged the stability with $P_{0}^{*}$ at *η*=*η*_*c*_ via the transcritical bifurcation, thus becoming globally stable. As mentioned, the increase of *η* involves the motion of $P_{2}^{*}$ towards higher population values, meaning that the SYN strain populations dominate over the WT ones. The results of the analysis reveal that, provided that the resource is not scarce, we just need a slight advantage of the engineered strain to make it successful and reaching a high population. Moreover, if the resource declines over time, *S*_w_ will remain high.

The potential relevance of this scenario is illustrated by the observation that pathogenic strains of *Vibrio* sp. might be a major player in the marine plastisphere (particularly plastic microplastic) as revealed by sequencing methods [74]. If this is the original (WT) strain, an engineered strain with no toxin genes and improved attachment to plastic substrates could be designed to replace the WT. Moreover, one could also consider a rather orthogonal scenario where the plastic garbage constitutes an opportunity for synthetic communities to thrive. Since plastic debris is known to be used by a large number of species as a stable substrate, it can be argued that it can be seen as an artificial niche that provides new opportunities for developing complex communities.

It should be noted that plastic degradation by micro-organisms is not necessarily good news: degradation (or accelerated fragmentation) of large plastic items leads to a faster transfer towards smaller plastic sizes, particularly microplastic that can be easily transferred to food webs [75]. Is removal of the human-generated waste a necessary condition for these designed motifs? An alternative terraformation approach could be using synthetic species that attach to the substrate without actively degrading it. The synthetic micro-organism could carry some beneficial function, such as providing useful molecules enhancing the growth or establishment of other species, thus again acting as ecosystem engineers.

Mounting evidence indicates that a rich community of species adapted to these substrates has been developing over the years. Metagenomic analyses indicate an enrichment of genes associated with surface-associated lifestyles [76]. Within a surrounding environment that is oligotrophic and species-poor, the plastic garbage defines a novel niche that has been fairly well colonized by a wide variety of species attached to the plastic substrate. In many cases, the resulting microbial community provides the scaffold for other species to thrive. Some proposals on using synthetic biology to address the problem of plastic garbage included a project aimed to facilitate the stable adhesion of plastic pieces with the goal of creating plastic islands (see [23] and references therein). In such a context, we could consider a TM where the colonization by a given species performing other functionalities could be designed, perhaps taking advantage of the niche as an opportunity to build a synthetic ecosystem.

#### 2.3.2 Sewage and landfill motifs

The last TM analysed is connected to a major class of waste generated by farming as well as by urban and, especially, by mega-urban areas associated with domestic, municipal and industrial sources. Urban centres incorporate massive infrastructures associated with the treatment of waste as an end part of the city metabolism [77]. Sewage systems offer a specially interesting opportunity to apply our approach. They contain large amounts of organic matter, along with a wide repertoire of molecules of different origins, from drugs to toxic chemicals. Because of the potential damage caused by organic matter-rich waters (which can promote blooms of heterotrophic organisms leading to oxygen depletion in rivers) sewage treatment deals with a combination of organic particles along with diverse filters and a treatment of the resulting sludge from anaerobic micro-organisms [78].

Similarly, landfills have been widely used as a cheap solution of storing waste, despite the environmental consequences involving pollution on a local scale associated with leaching as well as contributing to global warming due to methane emissions. Some other problems are related to the treatment of heavy metals, organic pollutants (particularly aromatic hydrocarbons) and other hazardous waste. In order to address these environmental problems, strains of micro-organisms could be used to target these molecules. This approach is known as bioremediation and has been used with different degrees of success [79,80]. In recent years, it has been suggested that the use of genomic search, along with systems and synthetic biology approximations should be considered as a really effective approach to this problem [27,81].

**Figure 10.**
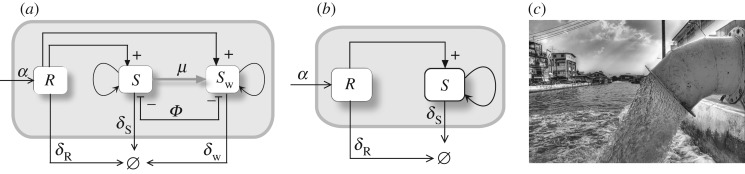
Terraformation motifs for the sewage and landfill systems. (*a*,*b*) A physical container (grey boxes) allows one to regulate input and output flows for these systems. In (*a*) the graph shows the resource-consumer structure of this motif, where both strains are supported by the same resource, while they compete and are connected through gene loss. A simpler alternative (*b*) does not require engineering of extant species since it is a completely artefactual ecosystem and its preservation is not required. A typical scenario would be sewage-related infrastructures (*c*, image obtained from Wikimedia Commons) where a rich microbial community is known to exist.

The basic designs, summarized in figure 10*a*,*b* (see also electronic supplementary material, Fig. S1d), incorporate some kind of ‘container’ indicating the presence of a physical boundary. This represents, for example, the sewage system of an urban centre or the spatial domain defining the limits of a landfill. In this way, we can define inputs and outputs associated with the inflow of water, organic matter or chemicals on the one hand and the outflow carrying other classes of molecules as well as micro-organisms on the other.

Here too it might be less relevant to preserve the existing species of microbes, given the less relevant motivation of preserving WT strains, thus making unnecessary to engineering from *S*_w_ (figure 10*b*). Of course, the real situation is more complex in terms of species and chemical diversity, and the single box indicating the resource *R* encapsulates a whole universe of chemical reactions. But we can also consider *R* as a very specific target for the SYN strain.

The mathematical model for the sewage and landfill motif (electronic supplementary material, Fig. S1d; and figure 10*a*) is given by the following system of nonlinear dynamical equations:

$$\begin{array}{rl} \frac{dR}{dt} & =\alpha -\delta_{R}R-\sigma_{S}RS-\sigma_{w}RS_{w},\\ \frac{dS}{dt} & =\eta \sigma_{S}RS+\rho_{S}S-\delta_{S}S-\mu S-S\Phi (\text{S}),\\ \frac{dS_{w}}{dt} & =\eta_{w}\sigma_{w}RS_{w}+\rho_{w}S_{w}-\delta_{w}S_{w}+\mu S-S_{w}\Phi (\text{S}). \end{array}$$

Here, as in the previous model, *α* and *δ*_*R*_ are, respectively, the rate of waste income and the spontaneous degradation rate of this resource. The degradation of the resource by the SYN and the WT strains is parametrized by *σ*_*S*_ and *σ*_w_, respectively. Some fraction of the degraded resource can be invested for growth and reproduction for both the SYN and the WT strains. The constants *η* and *η*_w_ parametrize these processes. Assuming the CP constraint, again with *S*+*S*_w_=1 and $\dot{S}+{\dot{S}}_{w}=0$, the outflow term is given by $\Phi (\text{S})=(\eta_{w}\sigma_{w}+\rho_{w}-\delta_{w})+S(\eta \sigma -\eta_{w}\sigma_{w}+\tilde{\rho }-\tilde{\delta })$. Using the previous conditions, the three-variable system is reduced to a two-dimensional dynamical system describing the dynamics of the resource (*R*) and the SYN strain (*S*), according to

2.4 $$\frac{dR}{dt}=\alpha -R[\delta_{R}+\sigma_{w}+\tilde{\sigma }S]$$

and

2.5 $$\frac{dS}{dt}=S[(1-S)(R(\tilde{\eta }(\sigma_{w}+\tilde{\sigma })+\eta_{w}\tilde{\sigma })+\tilde{\rho }-\tilde{\delta })-\mu ].$$

We note that here, for simplicity, we set $\tilde{\delta }=\delta_{S}-\delta_{w}$, $\tilde{\rho }=\rho -\rho_{w}$, $\tilde{\sigma }=\sigma_{S}-\sigma_{w}$ and $\tilde{\eta }=\eta -\eta_{w}$.

The fixed points for equations (2.4)–(2.5) are given by

$$P_{1}^{*}=\left(R_{1}^{*}=\frac{\alpha }{\delta_{R}+\sigma_{w}},S_{1}^{*}=0\right)$$

and the pair of fixed points $P_{\pm }^{*}$ (see electronic supplementary material, Section S4, for their values). The Jacobian matrix for the system above reads

2.6 $$J=\left(\begin{array}{cc} -\delta_{R}-\sigma_{w}-\tilde{\sigma }S & -R\tilde{\sigma }\\ S(1-S)\Theta & (1-2S)[R\Theta +\tilde{\rho }-\tilde{\delta }]-\mu \end{array}\right),$$

where $\Theta =[\tilde{\eta }(\tilde{\sigma }+\sigma_{w})+\eta_{w}\tilde{\sigma }]$.

The eigenvalues of the first fixed point, obtained from $\det|J(P_{1}^{*})-\lambda I|=0$, are given by

$$\begin{array}{rl} \lambda_{1} & =-(\delta_{r}+\sigma_{w}),\\ \lambda_{2} & =\tilde{\rho }-\tilde{\delta }-\mu +\frac{\alpha }{\delta_{R}+\sigma_{w}}\Theta . \end{array}$$

Note that *λ*_1_ is always negative, and thus the stability of $P_{1}^{*}$ entirely depends on *λ*_2_. From *λ*_2_, we can define the critical *μ* value:

$$\mu_{c}=\tilde{\rho }-\tilde{\delta }+\frac{\alpha }{\delta_{R}+\sigma_{w}}\Theta .$$

When *μ*>*μ*_*c*_, *λ*_2_<0 and thus *P*_1_ is stable. Under this stability condition the SYN strain will become extinct.

In order to focus on the most interesting parameters from the engineering point of view (i.e. $\tilde{\sigma }$ and $\tilde{\eta }$), we will hereafter take into account that both *S* and *S*_w_ strains reproduce at the same rates in the absence of *R* (i.e. $\tilde{\rho }=0$), also assuming that both strains have the same death rates (*δ*_w_=*δ*_*S*_, i.e. $\tilde{\delta }=0$). Under these assumptions, the equations now read

2.7 $$\frac{dR}{dt}=\alpha -R[\delta_{R}+\sigma_{w}+\tilde{\sigma }S]$$

and

2.8 $$\frac{dS}{dt}=S[(1-S)(R(\tilde{\eta }(\sigma_{w}+\tilde{\sigma })+\eta_{w}\tilde{\sigma }))-\mu ].$$

For the system above, the fixed point $P_{1}^{*}$ remains the same, but now there exists a single interior fixed point, given by $P_{2}^{*}=(R_{2}^{*},S_{2}^{*})$ with

$$R_{2}^{*}=\frac{\alpha \Theta +\mu \tilde{\sigma }}{(\delta_{R}+\sigma_{w}+\tilde{\sigma })\Theta }$$

and

$$S_{2}^{*}=\frac{\alpha \Theta -\mu (\delta_{R}+\sigma_{w})}{\alpha \Theta +\mu \tilde{\sigma }}.$$

The eigenvalues of the first fixed point, obtained from $\det|J(P_{1}^{*})-\lambda I|=0$, fixing $\tilde{\rho }=\tilde{\delta }=0$, are given by

$$\begin{array}{rl} \lambda_{1} & =-(\delta_{r}+\sigma_{w}),\\ \lambda_{2} & =\frac{\alpha }{\delta_{R}+\sigma_{w}}\Theta -\mu . \end{array}$$

As mentioned above, the stability of this fixed point will depend on *λ*_2_, and now the critical *μ* value involving a change in the stability of $P_{1}^{*}$ is given by

$$\mu_{c}=\frac{\alpha }{\delta_{R}+\sigma_{w}}\Theta .$$

**Figure 11.**
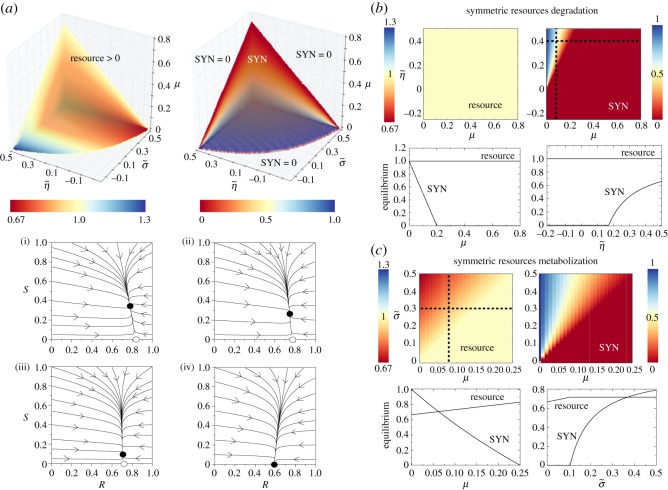
Representative dynamics of the sewage and landfill motifs. (*a*) Equilibrium population values (represented with a colour gradient) in the parameter spaces ($\tilde{\sigma },\tilde{\eta },\mu )$ obtained numerically from equations (2.7)–(2.8). We note that we plot the volume where the synthetic (SYN) strain survives and the corresponding resource equilibria for this volume (the resource is always present in the whole space). Below we display several phase plane portraits with: *a*(i) $\tilde{\sigma }=0.25$, *a*(ii) $\tilde{\sigma }=0.15$, *a*(iii) $\tilde{\sigma }=0.03$ and *a*(iv) $\tilde{\sigma }=-0.07$. Here, black and white circles mean stable and unstable fixed points, respectively. The arrows indicate the direction of the orbits. (*b*) Scenario with symmetric resource degradation for both strains, setting *σ*_w_=*σ*_*S*_≠0, i.e. $\tilde{\sigma }=0$. We display the equilibria for the resource and the SYN strain in the parameter space $\left(\mu ,\tilde{\eta }\right)$. Below we plot the bifurcation diagrams for the SYN strain and resource variables at equilibrium along the parameter values $\left(\mu ,\tilde{\eta }\right)$ indicated with the dashed lines in the panels above. (*c*) Scenario with symmetric resources metabolization *η*_w_=*η*≠0, with $\tilde{\eta }=0$. Here we also plot the population equilibria along the black dashed lines from the panels tuning *μ* and $\tilde{\sigma }$. In all of the analysis, we set *α*=1 and *δ*_*R*_=0.05.

Also, note that all the rest of the model parameters apart from *μ* are in *λ*_2_. Hence, the bifurcation can be also achieved tuning these parameters. For the case of *μ*, it can be shown that

$$P_{1}^{*}{|}_{\mu =\mu_{c}}=P_{2}^{*}{|}_{\mu =\mu_{c}}=\left(\frac{\alpha }{\delta_{R}+\sigma_{w}},0\right),$$

meaning that $P_{1}^{*}$ and $P_{2}^{*}$ collide at this bifurcation point, also exchanging their stability (see figure 11; electronic supplementary material, Fig. S4). Hence, we can conclude that this system suffers a transcritical bifurcation.

The impact of parameters $\tilde{\sigma }$, $\tilde{\eta }$ and *μ* on the equilibrium concentrations of the resource and the SYN strain is displayed in figure 11*a*. We note that the resource in the parameter space $(\tilde{\sigma },\tilde{\eta },\mu$) is always present. However, the boundaries causing extinction for the SYN strain are clearly seen (figure 11*a* (right)). These transitions are given by the transcritical bifurcation previously discussed. Similar to the previous models, the increase of *μ* involves the extinction of *S*, all the population being formed by the WT strain.

As expected, the increase of both *σ*_w_ and *η*_w_ also causes the extinction of the SYN strain. This actually means that if the WT has a fitness advantage in terms of resource degradation or metabolization, this population will outcompete the SYN strain. Note that this effect takes place when both $\tilde{\sigma }$ and $\tilde{\eta }$ decrease (i.e. *σ*_w_ and *η*_w_ increase, respectively), and the WT strain is able to degrade the resource faster or to better metabolize this resource. Under these conditions, and due to the competitive exclusion principle, the SYN strain will be outcompeted by the WT one. This process is accelerated at increasing parameter *μ*. The dynamics tied to different and decreasing values of $\tilde{\sigma }$ are represented in the phase portraits of figure 11*a*(i) and (iv).

Two specific cases can be considered here from the model given by equations (2.7)–(2.8) involving two different ecological scenarios that could be achieved by means of different engineering strategies. First, we will consider that both strains degrade the resource at the same rate (symmetric degradation) while their reproduction due to the consumption of such resource can be different. In the second scenario, we will consider that both SYN and WT strains degrade the resource at different rates but the metabolic efficiency of the resource is equal for both strains. Consider first the case where both the WT and the SYN strains degrade the resource at the same rates $\tilde{\sigma }=0$, with *σ*_w_≠0 and *σ*_*S*_≠0. However, their ability to metabolize the resource and use it for reproduction can be asymmetric (i.e. $\tilde{\eta }\neq 0$). The fixed points under these assumptions are given by $P_{1}^{*}$ (which remains the same as described above) and

$$P_{2}^{*}=\left(R_{2}^{*}=\frac{\alpha }{\delta_{R}+\sigma_{w}},\;S_{2}^{*}=1-\frac{\mu (\delta_{R}+\sigma_{w})}{\alpha \tilde{\eta }\sigma_{w}}\right).$$

Note that the transcritical bifurcation will take place when $S_{2}^{*}=0$ and when *λ*_2_=0. In particular, it can be shown that the SYN strain will survive when *μ*<*μ*_*c*_, with

$$\mu_{c}=\frac{\alpha \tilde{\eta }\sigma_{w}}{\delta_{R}+\sigma_{w}}.$$

The diagrams in figure 11*b* indicate such a smooth transition involving the extinction of the SYN strain. Together with *μ*, the other parameters involved in the transcritical bifurcation are *α*, $\tilde{\eta }$, *σ*_w_ and *δ*_*R*_. For instance, the bifurcation diagram tuning $\tilde{\eta }$ in figure 11*b* displays the transition at the value $\tilde{\eta }={\tilde{\eta }}_{c}$, with

$${\tilde{\eta }}_{c}=\frac{\mu (\delta_{R}+\sigma_{w})}{\alpha \sigma_{w}}.$$

As previously mentioned, the resource is always present, and its population is constant for this case since its equilibrium value does not depend on *μ* and $\tilde{\eta }$. Note that the coordinate $R_{2}^{*}$ is the same as $R_{1}^{*}$, which depend on parameters *α*, *δ*_*R*_ and *σ*_w_. This is the reason why the equilibrium of the resource does not change at the bifurcation. It is important to highlight that the concentration of the resources will decrease at increasing *σ*_w_. Hence, under symmetric degradation of the resources, the degradation efficiency of the WT will determine the equilibrium concentration of the resource. The corresponding bifurcation diagram of figure 11*b* shows the transitions of the SYN strain at *μ*=*μ*_*c*_ (left) and $\tilde{\eta }={\tilde{\eta }}_{c}$ (right).

Finally, we shall assume that both strains can degrade the resource differently $\left(\tilde{\sigma }\neq 0\right)$, but their efficiency to metabolize it (and thus reproduce) is the same, and thus $\tilde{\eta }=0$, with *η*≠0 and *η*_w_≠0. Under this scenario, the fixed points are again $P_{1}^{*}$ and $P_{2}^{*}$, $P_{2}^{*}$ now being given by

$$P_{2}^{*}=\left(\frac{\alpha \eta_{w}-\mu }{\eta_{w}(\delta_{R}+\sigma_{w}+\tilde{\sigma })},1-\frac{\mu \eta_{w}(\delta_{R}+\sigma_{w}+\tilde{\sigma })}{\alpha \eta_{w}-\mu }\right).$$

Here, the SYN strain will also survive when *μ*<*μ*_*c*_, now with

$$\mu_{c}=\frac{\alpha \eta_{w}\tilde{\sigma }}{\delta_{R}+\sigma_{w}}.$$

As shown in figure 11*c* the transition takes place at $\tilde{\sigma }={\tilde{\sigma }}_{c}$, with

$${\tilde{\sigma }}_{c}=\frac{\mu (\delta_{R}+\sigma_{w})}{\alpha \eta_{w}}.$$

Figure 11*c* shows that the equilibrium concentration of the resource depends on parameters *μ* and $\tilde{\sigma }$, although the concentration of the resource for the parameters analysed remains large. Increasing *σ*_w_ or *μ* involves the extinction of the SYN strain.

## 3. Conclusion

As our population rapidly increases our ecological demands also grow, pushing ecosystems to their limits. We probably move, thereby, to an uncertain future, where both ecosystems and societies could face the threat of catastrophic responses [82,83]. Humans have been highly successful as engineers, particularly as large-scale ecosystem engineers [84,85] since our activities have deeply modified the flows of matter and energy. To counterbalance runaway effects, we might need to develop new forms of ecological engineering. In this context, technological solutions grounded in geoengineering [86] have been suggested over the last decades [87–89] to counterbalance the damaging effects of rising carbon dioxide levels. The bioengineering alternative [23] offers a rather different (but perhaps complementary) path, where the technological solution has the potential for self-replication and thus for scaling up to the ecosystem-level requirements. However, a proper understanding of the implications and likelihood of these methods asks for a new synthesis of ideas and fields, from molecular biology to community ecology.

In this paper, we have mathematically explored four large classes of TMs that encapsulate different strategies. All of them involve engineering a given (microbial) species that is already present in the given ecological context. The proposed design goes beyond a standard bioremediation strategy, aiming at taking advantage of nonlinear responses, such as those displayed by (but not limited to) ecosystem engineers [90,91]. In this context, the proposed framework aims to extend the bioremediation picture [27] to larger scales and under a new set of ecological-grounded rules. Our models represent a first step in defining a population dynamics theory of synthetic ecosystems (considering the so-called TMs), that incorporate some classical modelling approaches with a constraint imposed by the nature of the synthetic component (which can revert to a WT strain). Our analysis provides a first glimpse of the potential relationships between key parameters allowing the SYN strain (and the implemented functionalities) to persist.

How reliable are our predictions? Several important factors have been left aside in our study, from diverse types of functional responses to stochasticity. However, as occurs with other nonlinear systems, we conjecture that some key results will not be strongly affected by the incorporation of additional nonlinearities or stochastic fluctuations. In this context, some fundamental types of bifurcations pervade the qualitative types of behaviours and parameter domain shapes reported above. The TMs including mutualistic interactions (studied in §2.2) revealed the presence of saddle-node bifurcations responsible for the extinction of both hosts and SYN strains. Interestingly, the replication rate of the SYN strain becomes a key parameter in allowing the persistence of the entire system. This phenomenon has been identified in all of the models including cooperation (§2.2). Our results indicate that restoration motifs operate gradually under transcritical bifurcations.

Because of the inevitable simplification imposed by our low-dimensional models, it can be argued that many potential biases might arise from diversity-related factors and spatial effects. Community dynamics might limit or even prevent the spread of the engineered strain, but we also need to consider how the changes derived from the engineering can propagate through the system (e.g. by horizontal gene transference). However, indirect evidence from manipulation experiments suggests that inoculation of micro-organisms can successfully change the organization and functionality of a given ecosystem in predictable ways, particularly in relation to soil crust ecosystems [32,59,92–95]. Future work should experimentally validate the predictions made here and further explore the limitations and potential extensions of our formalism.

## Supplementary Material

Supplementary Information

## Data accessibility

All the needed methods and results to replicate this article are provided as part of the paper and the electronic supplementary material.
