# Supplementary material for: Population dynamics of synthetic terraformation motifs
Source: R Soc Open Sci. 2018 Jul 4;5(7):180121. doi: 10.1098/rsos.180121 (PMC6083676; doi:10.1098/rsos.180121)
Supplement: Supplementary Information [file rsos180121supp1.pdf]

Population dynamics of synthetic terraformation motifs

Supplementary Information

Ricard Solé[1,2,3,*], Raúl Montañez[1,2], Salva Duran-Nebreda[1,2], Daniel Rodriguez-Amor[1,2,4], Blai Vidiella[1], Josep Sardanyés[1,2,5,6],

1. ICREA-Complex Systems Lab, Department of Experimental and Health Sciences, Universitat Pompeu Fabra, Dr. Aiguader 88, 08003 Barcelona
2. Institut de Biologia Evolutiva (CSIC-Universitat Pompeu Fabra), Pg. Maritim de la Barceloneta 37, 08003 Barcelona
3. Santa Fe Institute, 1399 Hyde Park Road, Santa Fe, NM 87501, USA
4. Physics of Living Systems, Department of Physics, Massachusetts Institute of Technology, Cambridge, Massachusetts, USA
5. Centre de Recerca Matemàtica. Edifici C, Campus de Bellaterra 08193, Bellaterra, Barcelona
6. Barcelona Graduate School of Mathematics (BGSMath). Edifici C, Campus de Bellaterra 08193, Bellaterra, Barcelona

$*$ corresponding author

Contents

1. Population dynamics of wild-type and synthetic strains 1
  1. Dynamics with mutation 1
  2. Dynamics with mutation and competition 2

2. Direct cooperation motifs: fixed points $P^*_\pm$ 4

3. Indirect cooperation motifs: fixed points and Jacobian matrix 4

4. Sewage and landfill motifs: fixed points $P^*_\pm$ 5

1. POPULATION DYNAMICS OF WILD-TYPE AND SYNTHETIC STRAINS

1. Dynamics with mutation

The dynamics for the wild-type (WT) and the synthetic (SYN) only considering mutation or gene loss resulting in the reversion to the WT species is given by the next couple of linear differential equations:

$$\begin{aligned} \frac{dS}{dt} &= \rho(1-\mu)S, \\ \frac{dS_w}{dt} &= \rho_w S_w + \mu\rho S. \end{aligned}$$

Here S and S_w are the population numbers of the SYN and the WT strains. Parameters ρ and ρ_w denote the replication rate of the SYN and the WT strains, while μ is the rate at which the genetic construct introduced in the SYN is lost, reverting to the WT.

Since the first equation is decoupled from S_w, it follows a simple linear growth with S and thus gives an exponential form:

$$S(t) = S(0)\exp\left(\rho(1-\mu)t\right).$$

Which now can be introduced into the differential equation for S_w and give:

$$\frac{dS_w}{dt} - \rho_w S_w = \mu\rho S(t),$$

where the Right-Hand Side (RHS) of this equation is replaced by the exponential form. The resulting equation can be solved and gives the solution:

$$S_w(t) = e^{\rho t}\left[\rho\mu S(0)\int e^{\rho(1-\mu)\tau}e^{-\rho_w\tau}d\tau + C\right],$$

C being the integration constant. The general solution gives:

$$S_w(t) = \xi e^{\rho(1-\mu)t} + (S_w(0)-\xi)e^{-\rho_w t},$$

where $\xi = \mu\rho S_0/(\rho(1-\mu)-\rho_w)$.

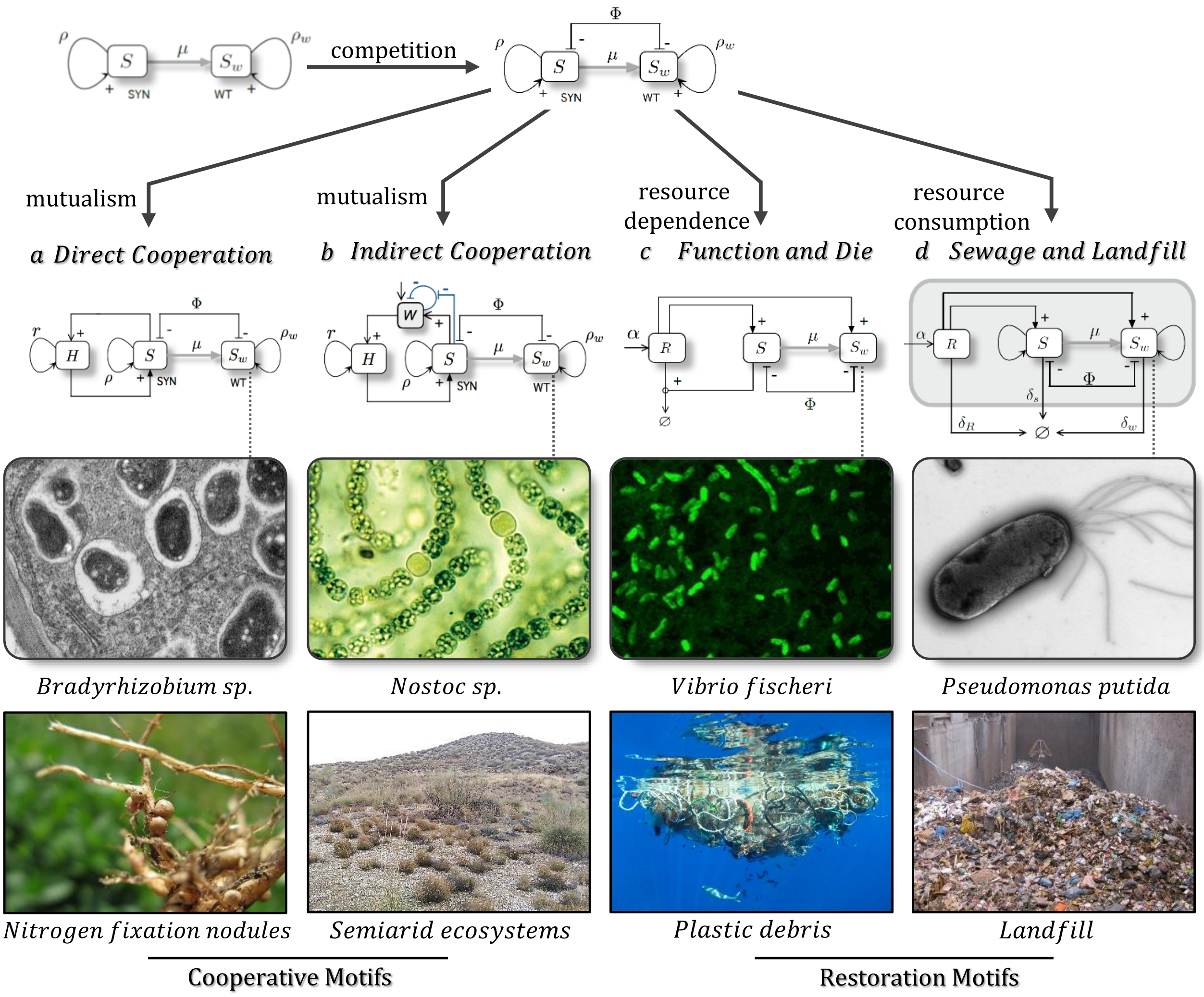

FIG. 1: Schematic diagram of the investigated synthetic Terraformation Motifs (TMs): mutualistic direct (a) and indirect (b) cooperation; function-and-die (c); and sewage and landfill (d) TMs. The two small diagrams above correspond to the wild-type (WT, S_w) and to the the engineered synthetic (SYN, S) strains. We assume that the SYN strain can loss the genetic instruct at rate μ reverting to the WT. Competition between the WT and the SYN is also considered in the four TMs. Some examples of potential species for being engineered are shown below the TMs. The lower panels display, for each TM, a model ecosystem that could benefit from the functions performed by the designed motifs. In (a) we display the nitrogen fixation nodules which enhance plants growth. The mutualistic motif represented in (b) could benefit semi-arid ecosystems by e.g., the synthesis of extracellular polymers able to retain water. The function-and-die motif involves the death of the SYN strain once the resource is completely degraded (illustrated by the degradation of plastic debris). In the sewage and landfill motif the resource is used for strains growth, although they can also reproduce without the presence of the resource. These motifs can be generally classified into cooperative (a-b) and restoration (c-d) motifs. Images obtained from Wikimedia Commons.

2. Dynamics with mutation and competition

The model with mutation analysed in Section 2.1.1 in the main manuscript (see also the section above) can be extended introducing competition between both strains. The system with competition can be described by:

$$\frac{dS}{dt} = \rho(1-\mu)S - S\Phi(\mathbf{S}),$$
$$\frac{dS_w}{dt} = \rho_w S_w + \mu\rho S - S_w\Phi(\mathbf{S}).$$

We are interested in the stability conditions associated with the two equilibrium (fixed) points of this system. From $dS/dt = 0$, we obtain $S_0^* = 0$ (extinction of the synthetic strain) and the non-trivial fixed point:

$$S_1^* = \left(1 - \frac{\mu\rho}{\rho - \rho_w}\right).$$

Using linear stability analysis, it is known that a fixed point S_k associated with a one-dimensional dynamical system $dS/dt = f(S)$ is stable provided that

$$\lambda(S_k) \equiv \left(\frac{df(S)}{dS}\right)_{S_k} < 0,$$

For our model we obtain $\lambda(S_k) = (\rho - \rho_w)(1 - 2S_k) - \mu\rho$, with $k = \{0, 1\}$. From the previous expression it can be seen that the fixed points exchange their stability when the critical condition $\mu\rho < \rho - \rho_w$ is fulfilled. In this context, the synthetic organism persists (i.e., S_1^* is stable) if μ is below a critical value μ_c, such as:

$$\mu < \mu_c = 1 - \frac{\rho_w}{\rho}. \quad (1)$$

Otherwise, it reverts to WT and gets extinct. This transition is given by a transcritical bifurcation i.e., the two fixed points exchange stability (the stable one becomes unstable and viceversa) when they collide at the value $S_0^*|_{\mu=\mu_c} = S_1^*|_{\mu=\mu_c} = 0$ (at the bifurcation point), being $\lambda(S_{0,1}^*)|_{\mu=\mu_c} = 0$. In this sense, when μ is increased, the fixed point S_1 moves towards the equilibrium point $S_0^* = 0$, colliding at the bifurcation point $\mu = \mu_c$. Similarly, from the stability condition Eq. (1) we can derive the critical values of ρ_c and ρ_w^c, given by $\rho_c = \rho_w/(1 - \mu)$, and $\rho_w^c = (1 - \mu)\rho$. Hence, the transcritical bifurcation will also take place at $\rho = \rho_c$ and $\rho_w = \rho_w^c$. In Fig. 3b in the main manuscript we show the nonlinear behaviour of this system for different values of the rate of reversion using ρ as control parameter. This is obtained by simply plotting S_1^* against mutation rate μ. Below the threshold, no synthetic organisms are viable, whereas for $\mu > \mu_c$ its population rapidly grows. This means that the competition is sharply resolved once we cross the critical rate μ_c.

In Fig. 3c (main manuscript) we also plot the associated phase diagram by defining the two main phases in the parameter space (ρ, μ). The persistence of the modified organism will be guaranteed (grey area) provided that it is either stable enough (low μ) or fast enough (high ρ) in replicating compared to the original strain. Inside Fig. 3c (main manuscript) we display the qualitative behavior of the flows on the line S (one-dimensional phase space) for each scenario. In the region where the synthetic consortium persists (grey area) the fixed point S_1^* is positive and stable (indicated with a black circle), while S_0^* is unstable (white circle). On the other hand, in the extinction scenario (white area), the fixed point S_1^* is negative and unstable, while the fixed point S_0^* is stable. At the critical boundary between survival and extinction both fixed points collide and become non-hyperbolic since $\lambda(S_{0,1}^*) = 0$.

This type of system is an example of competitive interactions incorporating a mutation term. The main lesson of this model is that a properly designed SYN organism such that it rarely reverts to the WT will expand and dominate the system, perhaps removing the WT. On the other hand, the SYN microbe must be capable of replicating fast enough to overcome the competition by the WT.

Now, we will investigate a slightly different model also describing competition between a SYN strain and the WT. The difference here is that the SYN strain will contain an engineered genetic construct that can be lost at a rate μ during the life of the strain. Hence, constant μ will be decoupled from replication. Now the model is given by:

$$\frac{dS}{dt} = S(\rho - \mu) - S\Phi(\mathbf{S}),$$
$$\frac{dS_w}{dt} = \rho_w S_w + \mu S - S_w \Phi(\mathbf{S}).$$

Here $\Phi(\mathbf{S})$ is again the outflow term. Following the previous procedure we can simplify the two-variable model to a one-dimensional dynamical system describing the dynamics of S, given by:

$$\frac{dS}{dt} = (\rho - \rho_w)S(1 - S) - \mu S.$$

This system behaves like the previous model: there are two equilibrium points that suffer a transcritical bifurcation once value of μ_c is achieved. The fixed points are now $S_0^* = (0)$, and the non-trivial one,

$$S_1^* = 1 - \frac{\mu}{\rho - \rho_w}.$$

The stability of S_0^* is given by $\lambda(S_0^*) = \rho - \rho_w - \mu$, while the stability of S_1^* is determined by $\lambda(S_1^*) = \rho_w - \rho + \mu$. From the previous values of λ we can compute the bifurcation value $\mu_c = \rho - \rho_w$. When $\mu < \mu_c$, S_1 is stable and S_0^* is unstable. At $\mu = \mu_c$ both fixed points collide interchanging their stability since $(S_0^*)|_{\mu_c} = (S_1^*)|_{\mu_c} = (0)$ and $\lambda(S_0^*)|_{\mu_c} = \lambda(S_1^*)|_{\mu_c} = 0$ (both equilibria are non-hyperbolic). For $\mu > \mu_c$, the fixed points S_0^* and S_1^* become, respectively, stable and unstable, meaning that the synthetic strain is outcompeted by the WT strain.

2. DIRECT COOPERATION MOTIFS: FIXED POINTS $P_{\pm}^*$

The fixed points $P_{\pm}^* = (H_{\pm}^*, S_{\pm}^*)$ of the models introduced in Section 2.2.1 (see main manuscript) are given by:

$$\begin{aligned} H_{\pm}^* &= \frac{1}{\eta(r+\gamma)}\Big[K\eta(r-\epsilon)+\gamma(\mu-\rho)+\frac{\beta}{2(K\eta+\rho-\rho_w)}+ \\ &+ \frac{K\eta[\rho(\gamma-r)+\rho_w(r-\gamma)]+\rho\rho_w(r-\gamma)}{K\eta+\rho-\rho_w}+\gamma\rho_w \pm \frac{1}{2}\sqrt{\Omega}\Big], \end{aligned} \quad (2)$$

with $\beta = K^2\eta^2(\gamma - r + \epsilon) + K\eta(\epsilon\rho - \gamma\mu - \epsilon\rho_w) + \gamma\mu(\rho_w - \rho) + \rho^2(\gamma - r) + \rho_w^2(\gamma - r)$, and $\Omega = -4\gamma(K\eta + \rho - \rho_w)(K\eta(\epsilon - r) + r(\mu - \rho + \rho_w)) + (K\eta(r - \gamma - \epsilon) + \gamma(\mu - \rho + \rho_w) + r(\rho - \rho_w))^2$.

The second coordinates of the fixed points $P_{\pm}^*$ read:

$$S_{\pm}^* = \frac{(K\eta(\gamma - r + \epsilon) + \gamma(\rho - \mu - \rho_w) + r(\rho_w - \rho) \pm \sqrt{\Omega})}{2\gamma(K\eta + \rho - \rho_w)}.$$

The fixed points setting $r = 0$ correspond to the dynamics with strict cooperation (Section 2.2.2 in the main manuscript). The case $r > 0$ corresponds to the model with facultative reproduction and cooperation (Section 2.2.3 in the main manuscript).

Numerical investigations carried out in Section 2.2.1 (main manuscript) indicated that the fixed point P_+^* is a stable node, being P_-^* a saddle (see Figs. 6 and 7 in the main manuscript). Both fixed points are created via a saddle-node bifurcation, and the node is the attractor responsible for the coexistence dynamics of the host and the SYN strains. The collision of the saddle and the node in the saddle-node bifurcation involves their annihilation and the jump of these fixed points into the complex phase space. In our case, we can compute the saddle-node bifurcation values by setting $\Omega = 0$ in Eq. (2). Under this condition, it can be shown that $P_-^*|_{\Omega=0} = P_+^*|_{\Omega=0}$. Hence, those parameters values making $\Omega = 0$ correspond to bifurcation values.

3. INDIRECT COOPERATION MOTIFS: FIXED POINTS AND JACOBIAN MATRIX

The fixed points P_4^* and P_5^* for the indirect cooperation model studied in Section 2.2.4 (main manuscript) are given by:

$$\begin{aligned} P_4^* : H &= \frac{a(-(S_c+1)\tilde{\rho}+\mu+(S_c+1)K\eta)\psi\beta^2 - K\epsilon(S_cLn+(S_c\tilde{\rho}+\tilde{\rho}-\mu)\psi)\beta+\sqrt{\theta}}{2(S_c+1)\beta\eta(a\beta+K\epsilon)\psi}, \\ S &= \frac{S_c\beta KL\eta\epsilon - \beta(a\beta((S_c-1)\tilde{\rho}+\mu+(S_c-1)K\eta)+K((S_c-1)\tilde{\rho}+\mu)\epsilon)\psi+\sqrt{\theta}}{2\beta(a\beta(\tilde{\rho}+K\eta)+\tilde{\rho}K\epsilon)\psi}, \\ W &= \frac{S_c\beta KL\eta\epsilon+\beta(a\beta(S_c\tilde{\rho}+\tilde{\rho}-\mu+(S_c+1)K\eta)+K(S_c\tilde{\rho}+\tilde{\rho}-\mu+2(S_c+1)K\eta)\epsilon)\psi+\sqrt{\theta})}{2\beta^2\psi(K(\tilde{\rho}-\mu+K\eta)\psi+c(\tilde{\rho}+K\eta)(L+K\psi))}, \\ &\text{and} \\ P_5^* : H &= -\frac{-a(-(S_c+1)\tilde{\rho}+\mu+(S_c+1)K\eta)\psi\beta^2+K\epsilon(S_cLn+(S_c\tilde{\rho}+\tilde{\rho}-\mu)\psi)\beta+\sqrt{\theta}}{2(S_c+1)\beta\eta(a\beta+K\epsilon)\psi}, \\ S &= -\frac{-c\beta KL\eta\epsilon+\beta(a\beta((S_c-1)\tilde{\rho}+\mu+(S_c-1)K\eta)+K((S_c-1)\tilde{\rho}+\mu)\epsilon)\psi+\sqrt{\theta}}{2\beta(a\beta(\tilde{\rho}+K\eta)+\tilde{\rho}K\epsilon)\psi}, \\ W &= \frac{S_c\beta KL\eta\epsilon+\beta(a\beta(S_c\tilde{\rho}+\tilde{\rho}-\mu+(S_c+1)K\eta)+K(S_c\tilde{\rho}+\tilde{\rho}-\mu+2(S_c+1)K\eta)\epsilon)\psi-\sqrt{\theta}}{2\beta^2\psi(K(\tilde{\rho}-\mu+K\eta)\psi+c(\tilde{\rho}+K\eta)(L+K\psi))}, \end{aligned}$$

with

$$\theta = \beta^2\left(S_c^2K^2L^2n^2\epsilon^2 - 2cKL\eta(a\beta(S_c\tilde{\rho}+\tilde{\rho}+\mu+(S_c+1)K\eta)+K(S_c\tilde{\rho}+\tilde{\rho}+\mu)\epsilon)\psi\epsilon+\right.$$

$$\left.+(a\beta(S_c\tilde{\rho}+\tilde{\rho}-\mu+(S_c+1)K\eta)+K(S_c\tilde{\rho}+\tilde{\rho}-\mu)\epsilon)^2\psi^2.$$

The Jacobian matrix for the three-variable model introduced in Section 2.2.4 (main manuscript) is given by:

$$\mathcal{J} = \begin{pmatrix} \frac{\beta\psi W(K-2H)}{K} - \epsilon & 0 & \beta H\psi\left(1-\frac{H}{K}\right) \\ -\eta(S-1)S & -2S(\tilde{\rho}+H\eta)+\tilde{\rho}+H\eta-\mu & 0 \\ -\psi W & \frac{S_cLW}{(S_c+S)^2} & -\frac{S_cL}{S_c+S} - H\psi \end{pmatrix}.$$

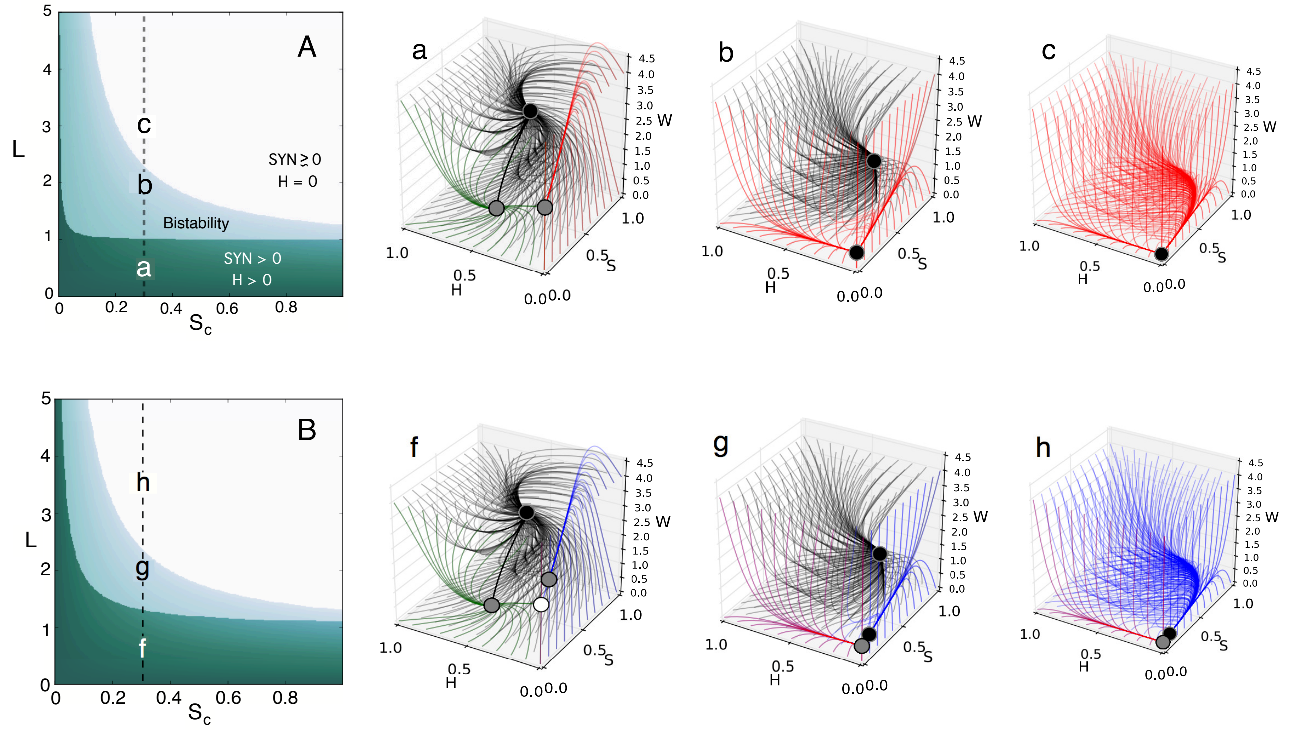

FIG. 2: Population equilibria and dynamics for the indirect cooperation motif depending on the the evaporation inhibition threshold (S_c) and on the evaporation rate (L). The parameters are the same used in Figure 7 in the main manuscript, setting $\rho = \eta = 1$. (A) Phase diagram with $\tilde{\rho} < \mu$, with $\rho_w = 0.91$. (B) Phase diagram with $\tilde{\rho} > \mu$, with $\rho_w = 0.89$. Three-dimensional phase portraits are displayed for three scenarios giving place to: monostable coexistence of the host with the WT and the SYN strains (a, f); coexistence under bistability (b, g); and extinction of vegetation (c, h).

4. SEWAGE AND LANDFILL MOTIFS: FIXED POINTS $P^*_\pm$

The pair of fixed points $P^*_\pm = (R^*_\pm, S^*_\pm)$ for the model describing the dynamics of the sewage and landfill motifs (see Section 2.3.2 in the main manuscript) are given by:

$$\begin{aligned} R^*_\pm &= \frac{1}{2\beta\xi}\Big[\delta_R(\tilde{\delta}-\tilde{\rho}) + \tilde{\sigma}(\tilde{\delta}+\alpha(\tilde{\eta}+\eta_w)+\mu-\tilde{\rho}) + \sigma_w(\tilde{\delta}+\alpha\tilde{\eta}-\tilde{\rho}) \\ &\mp \sqrt{(\delta_R(\tilde{\delta}-\tilde{\rho}) + \tilde{\sigma}(\tilde{\delta}+\alpha(\tilde{\eta}+\eta_w)+\mu-\tilde{\rho}) + \sigma_w(\tilde{\delta}+\alpha\tilde{\eta}-\tilde{\rho}))^2 - 4\alpha(\tilde{\delta}-\tilde{\rho})\beta\xi}\Big], \end{aligned}$$

and

$$\begin{aligned} S^*_\pm &= \frac{1}{2(\tilde{\delta}-\tilde{\rho})\tilde{\sigma}}\Big[\delta_R(\tilde{\rho}-\tilde{\delta}) + \tilde{\sigma}(\tilde{\delta}+\alpha(\tilde{\eta}+\eta_w)+\mu-\tilde{\rho}) - \sigma_w(\tilde{\delta}-\alpha\tilde{\eta}-\tilde{\rho}) \\ &\pm \sqrt{(\delta_R(\tilde{\delta}-\tilde{\rho}) + \tilde{\sigma}(\tilde{\delta}+\alpha(\tilde{\eta}+\eta_w)+\mu-\tilde{\rho} + \sigma_w(\tilde{\delta}+\alpha\tilde{\eta}-\tilde{\rho}))^2 - 4\alpha(\tilde{\delta}-\tilde{\rho})\beta\xi}\Big], \end{aligned}$$

with $\beta = \delta_R + \tilde{\sigma} + \tilde{\sigma_w}$ and $\xi = \eta_w\tilde{\sigma} + \tilde{\eta}(\tilde{\sigma} + \sigma_w)$.

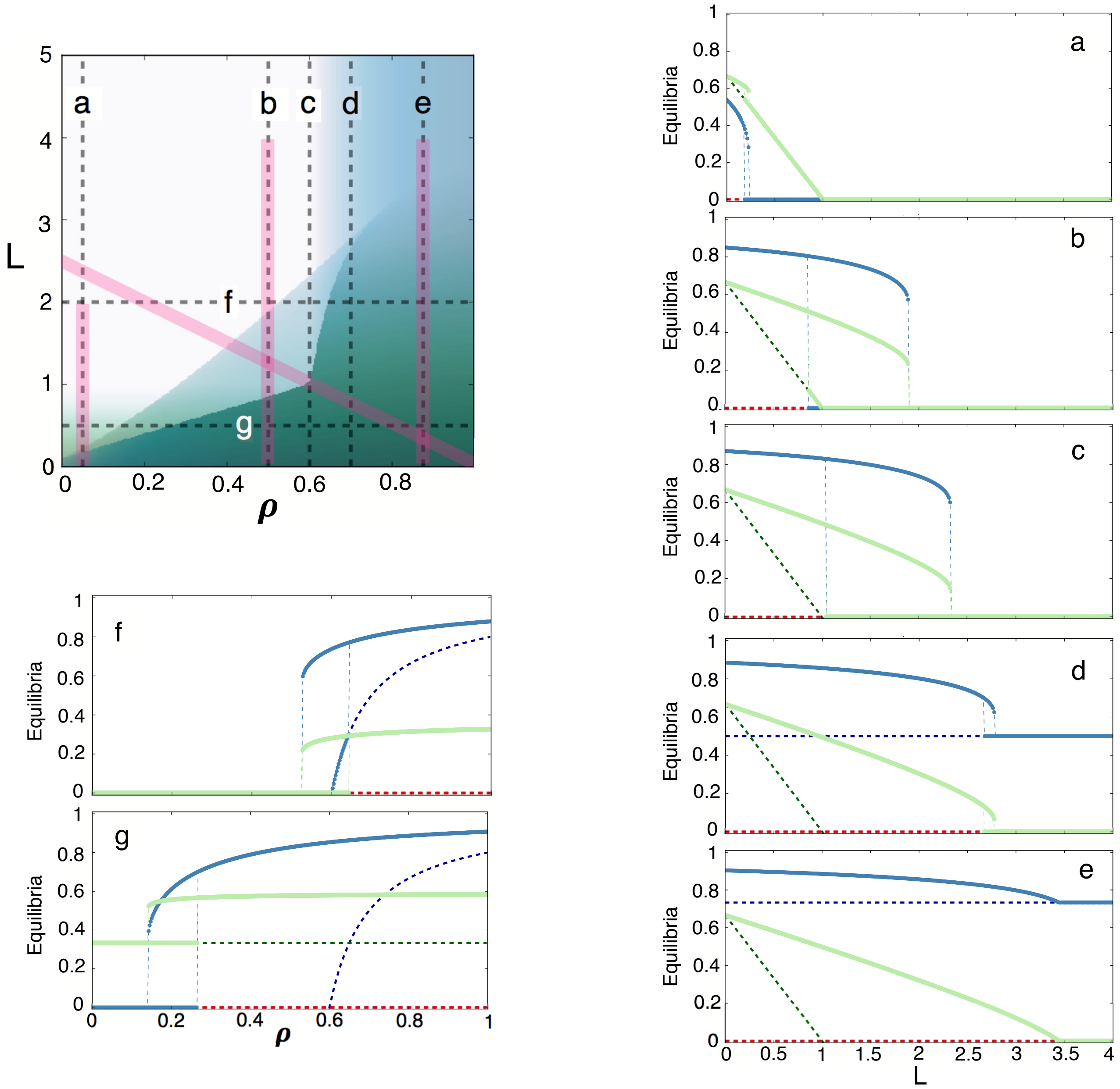

FIG. 3: Population equilibria for the indirect cooperation motif model (studied in Section 2.2.4 in the main manuscript), depending on the evaporation rate (L) and on the replication rate of the synthetic strain (ρ). The phase diagram is the same of Fig. 7B in the main article. The bifurcation diagrams (a-g) are built following the parameter ranges indicated with dashed lines in the phase diagram. Here we plot the numerical solutions of the synthetic strain S (light blue); and the vegetation (light green). The fixed points are indicated as follows: P_1^* (red), P_2^* (coordinate S, dark blue); and P_3^* (coordinate H, dark green). The thick pink lines correspond to the parameter values chosen to visualize the changes in the phase space, which are presented in the videos 1, 2, 3, and 4.

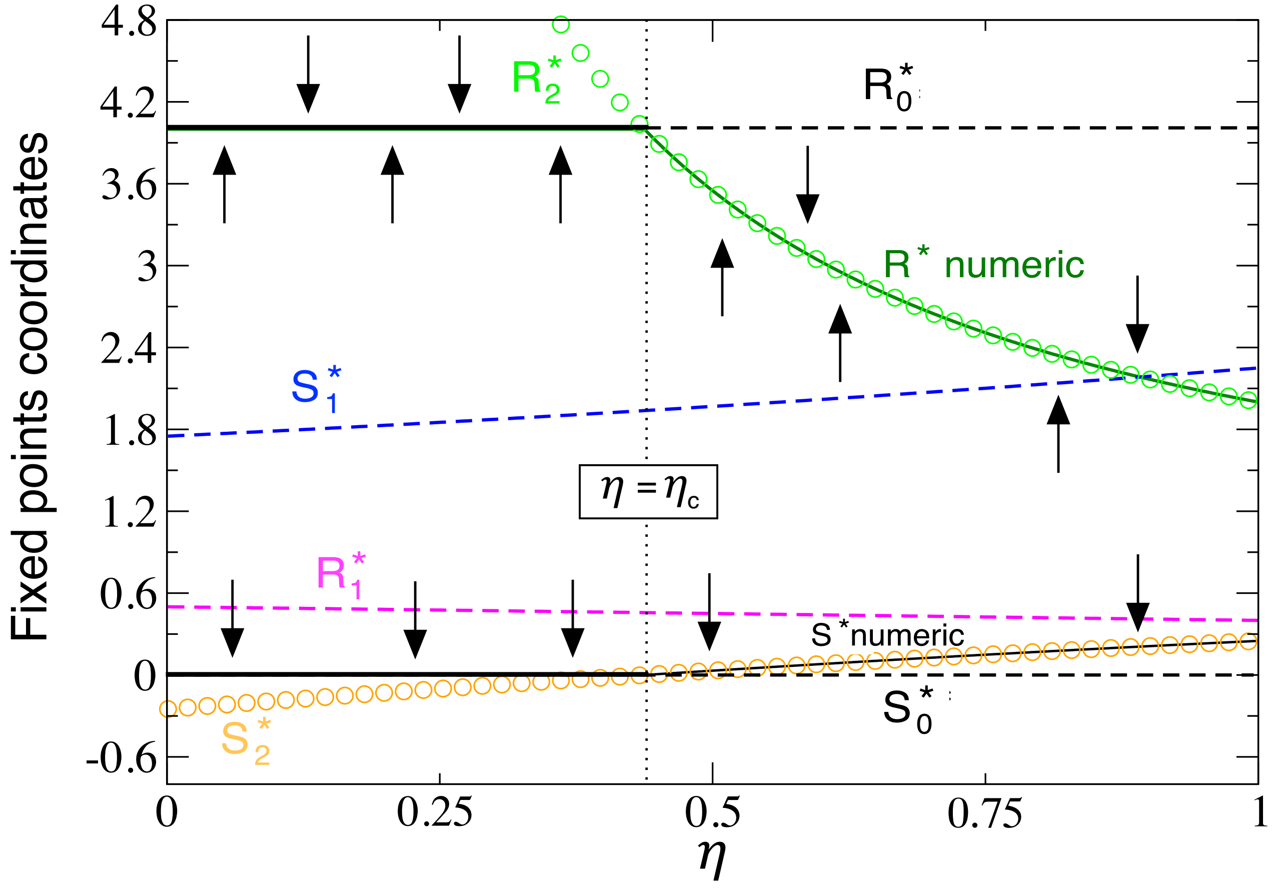

FIG. 4: Numerical representation of the coordinates for the fixed points $P^*_{0,1,2}$ for the *function-and-die motif* studied in Section 2.3.1 in the main manuscript. Here we reproduce the analysis of Fig. 9a (see main article) for the case $\mu = 0.75$, where parameter η is increased. Below its bifurcation value, $\eta < \eta_c$, the fixed point P^*_0 is stable (stable and unstable points are indicated, respectively, with solid and dashed lines). At $\eta = \eta_c = 0.4375$ (vertical dotted line) the fixed point P^*_2 collides with P^*_0, in a transcritical bifurcation where the stability between these two fixed points is exchanged. Then, for $\eta > \eta_c$, P^*_2 is stable and P^*_0 unstable, respectively. The coordinates R^*_2 and S^*_2 are represented, respectively, with green and orange circles. Note that overlapped to these coordinates we display the numerical solutions (black solid lines). The coordinates of P^*_1 are colored in magenta (R^*_1) and blue (S^*_1). Notice that the coordinate $S^*_1 > 1$ for all the values of η. The arrows indicate whether P^*_0 and P^*_2 are stable or unstable.
